# Major Trauma Triage Study (MATTS): Diagnostic accuracy of major trauma triage tools in English regional trauma networks – A case-cohort study

**DOI:** 10.1371/journal.pone.0344996

**Published:** 2026-03-27

**Authors:** Gordon Ward Fuller, James Baird, Samuel Keating, Joshua Miller, Richard Pilbery, Natalie Scotney, Katherine McKnee, Janette Turner, Fiona Lecky, Antoinette Edwards, Andy Rosser, Rachael Fothergill, Sarah Black, Fiona Bell, Michael Smyth, Jason E Smith, Gavin D Perkins, Stuart Reid, Esther Herbert, Stephen Walters, Cindy Cooper, and the MATTS research group

**Affiliations:** 1 School of Medicine and Population Health, School of Health and Related Research, Sheffield, South Yorkshire, United Kingdom; 2 CorEvitas, London, United Kingdom; 3 West Midlands Ambulance Service University NHS Foundation Trus, Brierley Hill, West Midlands, United Kingdom; 4 Yorkshire Ambulance Service NHS Trust, Wakefield, South Yorkshire, United Kingdom; 5 South Western Ambulance Service NHS Foundation Trust, Exeter, Devon, United Kingdom; 6 Trauma Audit and Research Network, University of Manchester, Manchester, United Kingdom; 7 London Ambulance Service NHS Trust, London, United Kingdom; 8 Clinical Trials Unit, University of Warwick, Coventry, United Kingdom; 9 Department of Emergency Medicine, University Hospitals Plymouth NHS Trust, Plymouth, United Kingdom; 10 Sheffield Teaching Hospitals NHS Foundation Trust, Sheffield, United Kingdom; Jazan University College of Applied Medical Science, SAUDI ARABIA

## Abstract

**Background:**

Major trauma is a leading cause of death and disability. Specialised care in major trauma centres has been associated with improved outcomes and prehospital triage tools are used to ensure injured patients are treated in the right place and the right time. However, there is a trade-off between under- and over-triage, and this study aimed to externally validate current and newly developed major trauma triage tools.

**Methods:**

A diagnostic case-cohort study was performed between November 2019 and February 2020 in 4 English regional trauma networks as part of the Major Trauma Triage Study (MATTS). The accuracy of 22 adult major trauma triage tools, including 3 newly developed MATTS tools was evaluated. Consecutive patients with acute non-trivial injury presenting to participating ambulance services were included and matched to data from the English national major trauma database. Theoretical accuracy was examined, with index tests assessed according to objective ambulance service data, regardless of the final triage decision or hospital destination. The primary reference standard was a consensus definition of serious injury that would benefit from expedited major trauma centre care.

**Results:**

The case-cohort sample consisted of 2,607 patients, including 928 primary reference standard positive patients. The population weighted prevalence of major trauma meeting the primary reference standard definition was 3.1% (95% CI 2.3–4.0). Four optimally performing triage tools were identified with Pareto decision analysis: the Trauma score (sensitivity 0.1, specificity 0.99), MATTS specific tool (sensitivity 0.37, specificity 0.95), MATTS balanced tool (sensitivity 0.58, specificity 0.87), and the MATTS sensitive tool (sensitivity 0.72, specificity 0.76). This finding was unchanged in subgroup analyses of different age-groups and injury mechanisms; secondary analyses examining alternative reference standards (ISS ≥ 16, US consensus definition); and sensitivity analyses exploring missing data.

**Conclusions:**

Four optimal triage tools, demonstrating a trade-off between sensitivity and specificity, were identified by this validation study. The choice of ideal tool will depend on prevalence of major trauma, and valuation of false positive and false negative cases. Further prospective investigation of real-life triage tool performance, including compliance and clinical judgment, is necessary.

## Introduction

Major trauma is an important public health issue. There are 20,000 cases annually in England, accounting for 5,400 deaths, 8,000 disabilities, and an estimated £400 million of health care costs [1]. The epidemiology of major trauma has evolved over recent decades, and it is now recognised to comprise two distinct disease entities: higher energy trauma in younger patients, and an increasing burden of low energy trauma in older patients (the ‘silver tsunami’) [2].

Major trauma care in England, and other developed countries, is organised into trauma networks, aiming to transport patients injured within a defined geographical area to a hospital matched to their clinical need [3]. Trauma networks consist of a specialist major trauma centre (MTC) offering advanced specialist care for the most seriously injured patients, and several lower-level hospitals capable of caring for lower-acuity injuries. Specialist care in MTCs has been associated with improved outcomes, although the benefit in older patients is less certain [4,5]. Major trauma triage tools are used to identify appropriate patients for direct transfer to MTCs, potentially bypassing other hospitals, and to inform pre-alert calls to allow activation of MTC trauma teams for expedited resuscitation and assessment [6]. Further details on English major trauma services and triage are provided in the supplementary materials (S1 File).

A wide range of triage tools are available, varying in structure and content [7]. As variables and cut-off points are altered, there is a trade-off in accuracy, with specificity falling as sensitivity increases. The optimal compromise depends on the prevalence of major trauma in injured patients presenting to ambulance services, and the relative valuation of false positives and negatives. The American College of Surgeons Committee on Trauma have recommended a target sensitivity of 95% and specificity of 65–75% [8]. However, multiple economic evaluations have consistently demonstrated that high specificity should be prioritised for cost-effective triage [9–12].

A recent systematic review of triage tool performance found poor quality studies and varying accuracy, with sensitivity ranging from 10% to 100%, and specificity from 9% to 100% [7]. The Major Trauma Triage Tool Study (MATTS) was funded by the National institute of Health Research to develop a new triage tool for use in the English National Health Service (NHS), validate its performance against existing triage tools, and evaluate its performance after implementation [13]. The current study reports validation of existing triage tools and three new MATTS triage tools. Specific objectives were to describe the characteristics of injured patients presenting to English ambulance services, determine the diagnostic accuracy of selected adult triage tools (including newly developed MATTS triage tools) and to evaluate their net benefit compared to default strategies of bypassing/pre-alerting all or no patients.

## Methods

### Study design

A diagnostic case-cohort study was undertaken to evaluate the accuracy of representative adult major trauma triage tools, and externally validate newly developed MATTS expert consensus triage tools. Case-cohort studies are recommended for evaluation of diagnostic accuracy in low prevalence scenarios [14]. In this study design, a random sample of individuals from the study population are included irrespective of reference standard (‘sub-cohort’), together with all reference standard positive patients (‘cases’) [14,15].

### Setting

The study was undertaken in four individual English inclusive regional trauma networks: Birmingham, West Yorkshire, North West London, and Severn. These are predominantly served by four separate NHS ambulance services: West Midlands Ambulance Service University NHS Foundation Trust (WMAS); Yorkshire Ambulance Service NHS Trust (YAS); London Ambulance Service NHS Trust (LAS); and the South-Western Ambulance Service NHS Foundation Trust (SWAS) respectively. The study trauma networks were chosen as they are principally served by a single ambulance service, have a favourable record for data collection, and represent a diverse range of localities, demographic, socioeconomic and injury profiles.

### Reference standards

The primary reference standard against which triage tool outcomes were assessed was injured patients who would benefit from expedited MTC care, as characterised by a previously published MATTS consensus-based definition [16]. Several alternative reference standards were considered in secondary analyses, including injury severity score (ISS) ≥16 [17], urgent trauma interventions, and the MATTS reference standard excluding open fractures. Urgent interventions comprised advanced airway interventions; transfusion of >4 units blood products; interventional radiology and/or emergency surgery occurring within 8 hours of emergency department (ED) arrival; ‘hot’ secondary transfer to the MTC within 6 hours, or critical care admission occurring within 12 hours of initial ED presentation.

### Index tests

The index tests under consideration were three newly developed MATTS expert consensus triage tools and 19 representative international and UK adult major trauma triage tools published prior to 2019, as detailed in Table 1. Development of the MATTS triage tools has been reported in detail elsewhere and constituent variables are presented in the supplementary materials (S2 Table) [18]. Theoretical diagnostic accuracy was examined, with index tests assessed according to objective data present in ambulance service records, regardless of the final triage decision or hospital destination. Discretionary variables were excluded. Prediction models and clinical scores were evaluated at their recommended published threshold. For assessment of the secondary MATTS reference standard excluding open fractures, triage tool variables for open fractures were omitted.

**Table 1:** Included triage tool index tests.

Number	Tool Abbreviation	Tool Full Name
1	CRAMS	Circulation, Respiration, Abdomen, Motor, and Speech Scale
2	Dutch	Dutch Field Triage Protocol
3	Florida	State of Florida Trauma Criteria
4	LAS (current)	London Ambulance Service (current) Major Trauma Triage Tool
5	LAS (old)	London Ambulance Service (old) Major Trauma Triage Tool
6	MATTS balanced	Newly developed MATTS triage tool – balancing sensitivity/specificity
7	MATTS sensitive	Newly developed MATTS triage tool – prioritising sensitivity
8	MATTS specific	Newly developed MATTS triage tool – prioritising specificity
9	MGAP	Mechanism, Glasgow Coma Scale, Age, and Arterial Pressure Score
10	North Carolina	North Carolina Trauma and Burn EMS Triage and Destination Plan
111	Oregon	Oregon Guidelines for Field Triage of Injured Patients
12	PHI	The Prehospital Index
13	RTST	Triage Revised Trauma Score
14	SWAS	South West Ambulance Service Major Trauma Triage Tool
15	Trauma Score	Trauma Score
16	Trauma Scorecard	Trauma Scorecard
17	TTR	Trauma Triage Rule
18	US Field Triage	National Guidelines for the Field Triage of Injured Patients (2011)
19	Victoria	Pre-hospital Major Trauma Triage – Trauma Victoria
20	Vittel	Vittel criteria for severe trauma triage
21	WMAS	West Midlands Ambulance Service Major Trauma Triage Tool
22	YAS	Yorkshire Ambulance Service Major Trauma Triage Tool
		

### Study population

The study sample was defined from the viewpoint of the ambulance service clinician assessing undifferentiated patients following injury where a major trauma triage tool would plausibly be used, thus providing the true denominator for assessment of diagnostic accuracy. The source population was all patients presenting with acute non-trivial injury to the four participating ambulance services and included trauma networks. The subsequent study population consisted of consecutive adult patients, aged over 16 years, conveyed to a participating trauma network hospital, between 1^st^ November 2019 and 28^th^ February 2020 and meeting study inclusion and exclusion criteria as detailed in Table 2. The final study sample included patients with complete data available allowing calculation of triage tool diagnostic accuracy.

**Table 2:** Inclusion and exclusion criteria.

Inclusion criteria	Exclusion criteria
Direct conveyance from scene to a hospital in included trauma network	Conveyed to non-participating hospital
Conveyed by participating ambulance service	Conveyed by non-participating ambulance service
Presented during 4 months study period	Not conveyed to hospital
Any age	Non-ambulance presentation
Presented with acute injury to ambulance services (selected WI code, major trauma pre-alert, trauma-specific intervention)	Death in field
Major trauma triage tool would plausibly be used	Secondary transfer
	Presentation out of study dates
	Trivial injury presentation (superficial bruises, abrasions, small uncomplicated lacerations, isolated joint sprains)
	Non-acute injury presentation (>72 hours)
	Medical presentation
	Isolated hypoxic injury (hanging, drowning)
	Isolated burns
	Triage tool exclusions: Traumatic cardiac arrest, Unstable ABC/divert to TU

### Case-cohort derivation

#### Sub-cohort identification.

Consecutive patients presenting with injury were identified from YAS, WMAS, LAS and SWAST patient records. Working impression diagnostic codes corresponding to non-trivial injury, use of a trauma-specific intervention, or major trauma related pre-alerts were selected for inclusion. Ambulance service databases were searched for any cases meeting these criteria who were injured in the defined study area and transported directly to a participating hospital to give the parent cohort. A random sample of patients were then included in a sub-cohort.

#### Reference standard positive case identification.

The presence of major trauma in patients conveyed to participating hospital by ambulance following injury was determined using Trauma Audit and Research Network (TARN), the English national major trauma registry, data [19,20]. Reference standard criteria were applied to all cases submitted to TARN and positive cases then linked to ambulance service data. A census sample of all cases conveyed to a selected trauma network hospital during the study period by a participating ambulance service, meeting ambulance service inclusion criteria and reference standard definitions, and matching to an ambulance service case were included. Any included ambulance service cases without a corresponding TARN submission were assumed to be reference standard negative.

Data linkage between a) cases meeting reference standard criteria collected by TARN, and b) ambulance identified patients with non-trivial injuries was conducted deterministically based on a unique ambulance service patient report form number shared across both datasets. In cases where exact deterministic matching was not possible due to missing or inaccurate patient report form number, research paramedics performed probabilistic matching by manually reviewing each reference standard positive case. Demographic (age, sex), non-unique ambulance identifiers (computer aided dispatch numbers, ambulance call sign) and incident information (incident date/time, hospital destination, hospital arrival date/time, incident description, incident location (outward and partial inward postcode)) was used from TARN data to search for a corresponding record in ambulance service databases. All matches were independently confirmed by a second researcher and a match was not confirmed in the presence of any uncertainty or disagreement.

### Data collection

#### Index test data.

Ambulance service records for patients sampled in the sub-cohort, or matched to non-sampled reference standard positive cases, were imported into a bespoke research database. Collected information comprised demographic, patient characteristics, physiology, incident, mechanism of injury, interventions, treatments, and clinical assessment information. Relevant electronic closed field data were imported directly where possible, with free text data coded by hand after review by research paramedics. Eligibility against inclusion and exclusion criteria was confirmed manually. Data abstraction was blinded, with all ambulance service data anonymised and reference standard status not available. A data dictionary was developed *a priori to* standardise all data elements, provide variable definitions, and define categorisations. Data collection was piloted, and weekly meetings were convened to review data collection, with any uncertainties resolved through consensus. Data was recorded as missing if not present in closed fields, or if not possible to infer from free text fields. Triage tools and prediction models selected as index tests were then coded algorithmically according to their stated variable thresholds against the observed data. The first recorded vitals sign was used for physiology variables. Where triage tool variables were defined as sustained physiology values, two or more consecutive values meeting the threshold were required. Two independent statisticians undertook coding to ensure accuracy.

#### Reference standard data.

TARN is the English national trauma registry database and collects information on patients with major injuries presenting to all trauma receiving hospitals in England. TARN data collection has been described in detail previously [21]. Briefly, patients are included in the TARN database if they sustain injury resulting in any of: hospital admission for >72 hours; critical care or high dependency unit admission; transfer for specialist care; or death. Patients with simple isolated closed injuries (e.g., radius fracture), pubic rami or femoral neck fractures and aged over 65, pre-hospital deaths, or diagnosed as dead on emergency department arrival with no further management instigated, are excluded. TARN data collectors in member hospitals collate demographic, injury, treatment, investigation, and outcome data for eligible patients from all relevant patient records. Independent, trained, TARN injury coders centrally grade individual injuries for each case according to the Abbreviated Injury Scale (AIS 2008 revision) criteria based on clinical, radiological and post mortem information. From the AIS severity scores a subsequent ISS is assigned [22].

Each submitted TARN case from participating hospitals during the study period was coded centrally by TARN data analysts according to primary and secondary reference standard criteria. Anonymised data for reference standard positive cases were then imported into a study database for review by research paramedics. Eligibility against inclusion and exclusion criteria was confirmed manually.

### Statistical analyses

The analysis proceeded in 5 stages. Firstly, the derivation of study population, parent cohort, sub-cohort, and reference standard positive cases were enumerated and delineated graphically using flow charts. Secondly, the study sample was characterised, with patient demographics, injury features and missing data examined using descriptive statistics. Thirdly, in the main analysis, the diagnostic accuracy of individual adult triage tools was investigated against primary and secondary reference standards. Sensitivity, specificity, positive and negative predictive values, and likelihood ratios were calculated with their 95% confidence intervals. Results were displayed graphically using diagnostic forest plots and plots of individual triage tools paired sensitivity/specificity. Separate sensitivity/specificity plots were constructed to present the Pareto front (or receiver operating characteristic curve convex hull), including only non-dominated triage tools with higher specificity for any given sensitivity [23–25]. The Pareto front was formed by linear interpolation between adjacent points with no point lying above the final curve. A decision curve analysis was then performed to illustrate the clinical consequence of implementing Pareto optimal triage tools [26]. Clinical net benefit of the identified tools was compared to default strategies of bypassing/pre-alerting all or no patients. Net benefit was calculated across a range of threshold probabilities, defined as the minimum probability of major trauma at which bypass/pre-alerting would be warranted, calculated as net benefit = sensitivity × prevalence – (1 – specificity) × (1 – prevalence) × the odds at the threshold probability [27].

Fourthly, subgroup analyses were conducted examining triage tool performance against the primary reference standard for adults (aged 16–64 years) and elderly patients (aged 65 years or older), and for cases with penetrating versus blunt injury, and higher energy injury presentations versus ground level falls. These were chosen as clinically relevant subgroups where previous studies have reported heterogenous triage tool performance.

Fifthly, sensitivity analyses were performed to explore the influence of study selection criteria and missing data. The main analysis was repeated, including primary reference standard positive cases involved in acute non-trivial injury events, but where a non-selected working impression code had been used. The main analysis was also repeated including eligible patients with missing data under a range of missing data mechanism assumptions. To indicate the range of possible results, best- and worst-case scenario analyses were conducted under a missing not at random assumption, where eligible patients with missing data were classified either as triage tool positive, or triage tool negative respectively. Multiple imputation using chained equations was also performed to explore the impact of data being missing at random [28].

Analyses were conducted in R Statistical Software (v4.3.0; R Core Team 2023) and STATA version 17.0 (StataCorp. 2016. Stata Statistical Software: Release 17. College Station, TX: StataCorp LP). Multiple imputation was performed using the *ice* procedure and the *mim* command was subsequently used to calculate triage rule sensitivity and specificity, combining results from imputed data sets according to Rubin’s rules. Unweighted summary statistics were reported separately for the sub-cohort and case-cohort sample characteristics and reference standard prevalence. Diagnostic accuracy metrics independent of underlying prevalence (sensitivity, specificity, likelihood ratios) were also calculated without sample weights. Conversely, Borgan II inverse probability sampling weights were used for positive and negative predictive values to represent non-sampled non-trivially injured patients, and account for the case-cohort study design [29]. The unit of analysis was the individual incident. Direct patient identifiers were unavailable, and it was therefore not possible to account for clustering from in the event recurrent incidents in the same patient.

### Sample size calculation

An estimated 600 reference standard positive patients were expected across the four participating ambulance service during the four-month study period. Assuming, based on ACS-COT (American College of Surgeons Committee on Trauma) criteria,[19] a triage tool required sensitivity of 95%, and anticipating that up to 5% of cases would have insufficient data, a sample of 570 cases would result in a 95% confidence interval (CI) width of ±2.0%. According to ACS-COT criteria a triage tool requires a specificity of 70%. [19] Therefore, to achieve a 95% CI width of ±2.0% a sample 2,014 reference standard negative patients would be required. This sample size would provide a 95% CI of ±5.0% and ±2% for under-triage rate in paediatric and elderly subgroups respectively.

### Ethical approval and data governance

This study was undertaken as part of the Major Trauma Triage Study (MATTS) project, aiming to develop a new national triage tool [13]. Ethical approval was provided by Yorkshire and The Humber – Bradford Leeds Research Ethics Committee (Reference: 19/YH/0197). Approval to use anonymised patient data without direct patient consent was confirmed by the Health Research Authority Confidentiality Advisory Group in accordance with Regulation 5 of the English Health Service (Control of Patient Information) Regulations 2002 (Reference: 19/CAG/0119). A study protocol and statistical analysis plan were pre-specified.

## Results

### Sample derivation and characteristics

**Fig 1:**
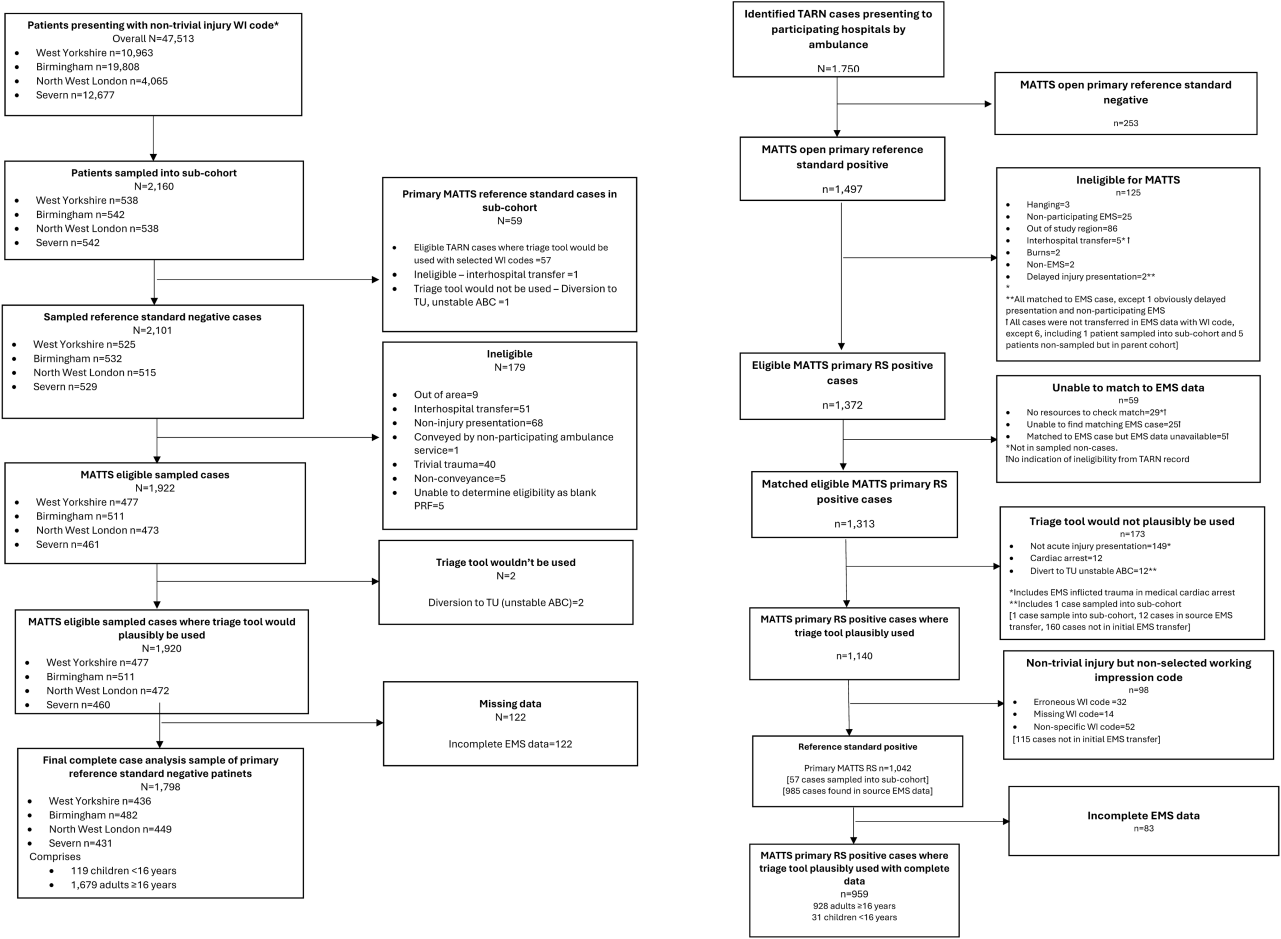
Derivation of primary reference standard positive and negative patients.

**Fig 2:**
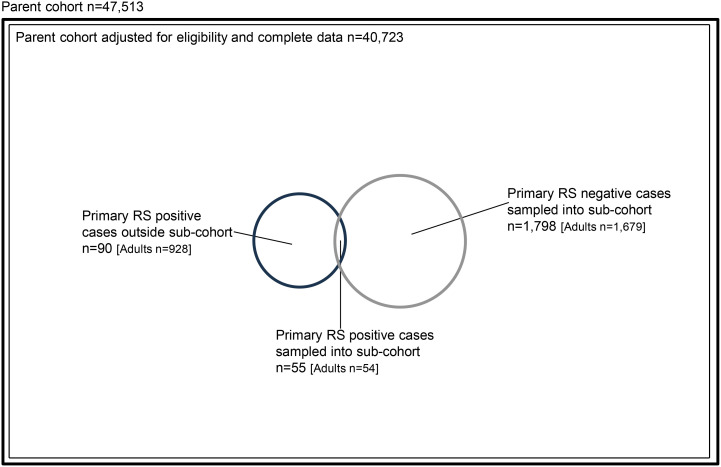
Case-cohort derivation of primary reference standard positive and negative cases in sub-cohort and parent cohort. Area of circles and squares is proportional to the number of patients..

Between 1st November 2019 and 28th February 2020, 47,513 patients with non-trivial injury working impression codes were conveyed to included trauma network hospitals by participating ambulance services, forming the source population. In total, 1,733 eligible adults with complete data were included in the sub-cohort, comprising 54 adults patients meeting primary reference standard criteria, and 1,679 adults who were primary reference standard negative. During the same study period 928 adult TARN cases met inclusion criteria, were successfully matched to a corresponding ambulance service record, and had complete data. Of these, 54 were included in the sub-cohort as described above, and 874 were outside the sub-cohort, but matched to ambulance cases in the parent cohort. The final case-cohort study sample for evaluation of adult triage tools therefore consisted of 2,607 patients (928 primary reference standard positive patients, 1,679 primary reference standard negative patients). Derivation of the parent cohort, sub-cohort, and study samples for primary reference standard positive and negative patients are detailed in Figs 1 and 2.

The overall prevalence of major trauma meeting the primary reference standard definition in eligible sub-cohort patients with complete data was 3.1% (n = 54/1,733 95% CI 2.3–4.0). Included patients presenting to ambulance services with non-trivial injury were predominantly elderly (median age 75 years), female (53.5%), and sustained accidental (88.1%) blunt trauma (97.7%) from ground level falls (71.2% of injury mechanisms). Characteristics of the included complete case study sample are detailed in Table 3.

**Table 3:** Characteristics of complete case case-cohort study sample.

Variable	Category	Reference standard positive (n = /928)	Reference standard positive summary statistic	Reference standard negative (n = /1,679)	Reference standard negative summary
**Demographics**					
Age (years)	Age	928	Median 62 IQR 41.5–79.5 Range 16–102	1,679	Median 76 IQR 47–86 Range 16–103
Age groups (%)	Adult 16–64.9 Elderly >65	491 437	52.9% 47.1%	644 1035	38.4% 61.6%
Sex (%)	Female Male	388 539	41.9% 58.1%	896 761	54.1% 45.9%
Residence (%)	No fixed abode Own home Rehabilitation centre Residential home Nursing home Other	5 850 4 12 3 54	0.5% 91.6% 0.4% 1.3% 0.3% 5.8%	17 1,385 7 147 103 12	1.0% 82.5% 0.4% 8.8% 6.1% 0.7%
**Injury characteristics**					
Mode of injury	Blunt Penetrating	873 55	94.1% 5.9%	1,641 38	97.7% 2.3%
Mechanism of injury	Cutting/piercing/stabbing Gunshot Fall > 1m Fall < 1m RTC (motorcycle) RTC (motor vehicle) RTC (bicycle) RTC (pedestrian) Struck by/collision with object Struck by/collision with person Other / unknown	54 1 228 335 39 75 29 95 41 19 12	5.8% 0..1% 24.6% 36.1% 4.2% 8.1% 3.1% 10.2% 4.4% 2.2% 1.3%	38 0 93 1,219 20 63 18 37 70 91 17	2.3% 0% 5.5% 72.6% 1..2% 3.8% 1.1% 2.2% 4.2% 5.4% 1.0%
**Vital signs**					
SBP	Median IQR Range	928	138 120-157 41-279	1,679	143 127-162 50-258
RR	Median IQR Range	928	20 17-22 0-56	1,679	18 16-20 11-67
Peripheral oxygen saturations	Median IQR Range	928	97 94-98 0-100	1,679	97 95-98 68-100
GCS	Median IQR Range	928	15 14-15 3-15	1,679	15 15−15 3-15
Pulse	Median IQR Range	928	85 72-100 25-178	1,679	83 72-95 38-180
**Incident characteristics**					
Call category	Category 1 Category 2 Category 3 Category 4 Category 4 Unknown	199 505 173 12 37 2	21.4% 54.4% 18.6% 1.3% 4.0% 0.2%	89 734 701 46 107 2	5.3% 43.7% 41.8% 2.7% 6.4% 0.1%
HEMS response	Yes No	173 755	18.6% 8.4%	22 1657	1.3% 98.7%
High priority leaving scene	Yes No	518 410	55.8% 44.2%	147 1532	8.9% 91.1%
Major trauma					
Injury Severity Score	Injury Severity Score	928	Median 18 IQR 14–25 Range 4–66		
Number of injured AIS body regions	1 2 3 4 5 6	326 304 193 81 16 9	35.1% 32.8% 20.7% 8.7% 1.7% 1.0%		
AIS body region injured	Head Face Thorax Abdomen Extremities External	591 134 379 189 468 307	52.9% 14.4% 40.8% 20.4% 50.4% 33.1%		
Open fracture (proximal to wrist/ankle)	No Yes	781 147	84.2% 15.8%		
Any urgent trauma intervention*	No Yes	664 284	69.4% 30.6%		

*GCS: Glasgow Coma Scale ISS: Injury severity score, RR: Respiratory rate, SBP: systolic blood pressure, RTC: Road traffic collision

*Comprises: MTP activation, > 4 units blood products, advanced airway intervention, interventional radiology, emergent trauma operation, emergency transfer to MTC, critical care admission.

### Main analysis

**Fig 3:**
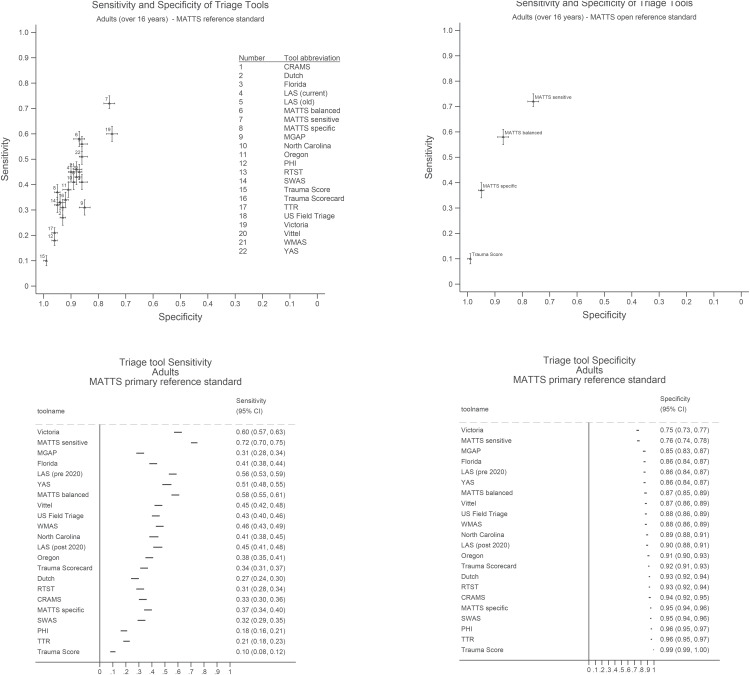
Sensitivity-Specificity plot for selected adult triage tools evaluated against the primary MATTS reference standard in patients aged over 16 years. Left panel shows all 22 triage tools. Right panel shows Pareto front of optimal tools, excluding tools with worse accuracy..

**Fig 4:**
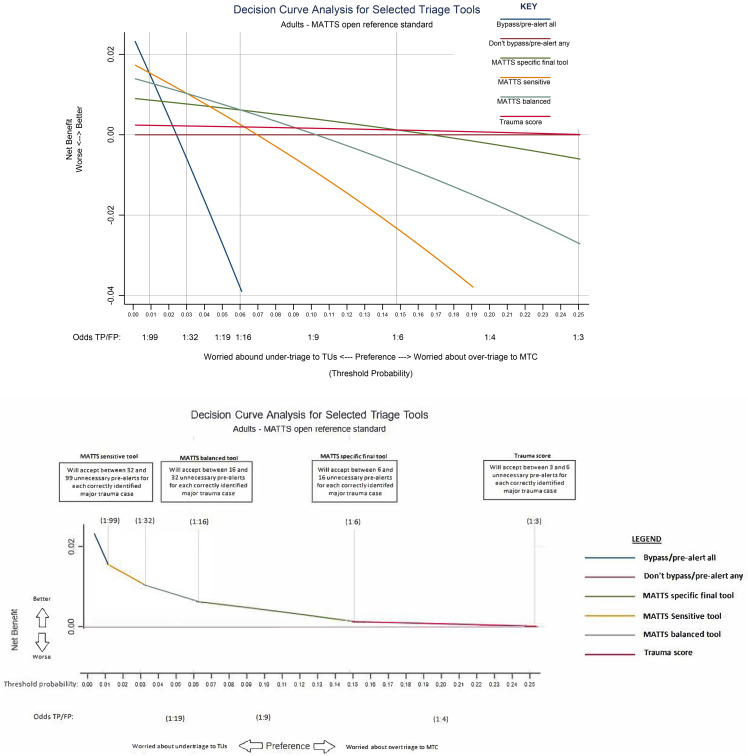
Decision curve analysis curves for Pareto optimal triage tools evaluated in adult patients against the primary reference standard. Top panel shows full decision curve. Bottom panel shows the frontier of optimal triage tools at different threshold probabilities.

In general, an inverse relationship between specificity and sensitivity for the primary reference standard was observed across triage tools, with an increasing false positive fraction as the proportion of false negatives decreased (Table 4, Fig 3). A Pareto front, with higher specificity for a given sensitivity, of four optimal triage tools was evident, comprising: Trauma score, MATTS specific tool, MATTS balanced tool, and the MATTS sensitive tool (Fig 3). Within this set of triage tools, the sensitivity/specificity trade-off varied between 0.10/0.99, 0.37/0.95, 0.58/0.87, to 0.72/0.76 respectively. The decision curve analysis indicated that the MATTS specific triage tool was the best choice if between 6 and 16 false positives would be accepted for each true positive case meeting the primary reference standard (Fig 4). Relative tool performance was similar when evaluated against secondary reference standards, with the same four triage tools (Trauma score, MATTS specific tool, MATTS balanced tool, MATTS sensitive tool) maximising accuracy and demonstrating Pareto dominance (see supplementary materials S3 Table).

**Table 4:** Diagnostic accuracy metrics for selected adult triage tools evaluated against the primary MATTS reference standard in patients aged over 16 years.

No.	Tool name	N	TP	FP	TN	FN	Sensitivity	LCL	UCL	Specificity	LCL	UCL	PPV*	LCL	UCL	NPV*	LCL	UCL	Positive LR	LCL	UCL	Negative LR	LCL	UCL
1	CRAMS	2607	310	107	1572	618	0.33	0.3	0.36	0.94	0.92	0.95	0.12	0.10	0.15	0.98	0.98	0.98	5.24	4.27	6.43	0.71	0.68	0.75
2	Dutch	2607	251	113	1566	677	0.27	0.24	0.3	0.93	0.92	0.94	0.09	0.08	0.12	0.98	0.98	0.98	4.02	3.27	4.94	0.78	0.75	0.82
3	Florida	2607	379	239	1440	549	0.41	0.38	0.44	0.86	0.84	0.87	0.07	0.06	0.08	0.98	0.98	0.98	2.87	2.49	3.30	0.69	0.65	0.73
4	LAS (current)	2607	414	176	1503	514	0.45	0.41	0.48	0.90	0.88	0.91	0.10	0.09	0.12	0.98	0.98	0.99	4.26	3.64	4.98	0.62	0.58	0.66
5	LAS (old)	2607	518	238	1441	410	0.56	0.53	0.59	0.86	0.84	0.87	0.09	0.08	0.11	0.99	0.99	0.99	3.94	3.46	4.49	0.52	0.48	0.56
6	MATTS balanced	2607	538	219	1460	390	0.58	0.55	0.61	0.87	0.85	0.89	0.10	0.09	0.12	0.99	0.99	0.99	4.45	3.88	5.09	0.48	0.45	0.52
7	MATTS sensitive	2607	672	407	1272	256	0.72	0.7	0.75	0.76	0.74	0.78	0.07	0.06	0.08	0.99	0.99	0.99	2.99	2.72	3.28	0.36	0.33	0.41
8	MATTS specific	2607	346	81	1598	582	0.37	0.34	0.40	0.95	0.94	0.96	0.17	0.14	0.21	0.98	0.98	0.99	7.73	6.15	9.71	0.66	0.63	0.69
9	MGAP	2607	289	253	1426	639	0.31	0.28	0.34	0.85	0.83	0.87	0.05	0.04	0.06	0.98	0.98	0.98	2.07	1.78	2.40	0.81	0.77	0.85
10	North Carolina	2607	385	181	1498	543	0.41	0.38	0.45	0.89	0.88	0.91	0.09	0.08	0.11	0.98	0.98	0.99	3.85	3.29	4.50	0.66	0.62	0.69
11	Oregon	2607	350	146	1533	578	0.38	0.35	0.41	0.91	0.9	0.93	0.10	0.09	0.13	0.98	0.98	0.98	4.34	3.64	5.17	0.68	0.65	0.72
12	PHI	2607	168	66	1613	760	0.18	0.16	0.21	0.96	0.95	0.97	0.11	0.08	0.14	0.98	0.98	0.98	4.61	3.50	6.05	0.85	0.83	0.88
13	RTST	2607	289	121	1558	639	0.31	0.28	0.34	0.93	0.92	0.94	0.10	0.08	0.13	0.98	0.98	0.98	4.32	3.55	5.26	0.74	0.71	0.78
14	SWAS	2607	300	80	1599	628	0.32	0.29	0.35	0.95	0.94	0.96	0.15	0.12	0.19	0.98	0.98	0.98	6.79	5.37	8.57	0.71	0.68	0.74
15	Trauma Score	2607	92	14	1665	836	0.10	0.08	0.12	0.99	0.99	1.00	0.26	0.15	0.40	0.98	0.98	0.98	11.89	6.82	20.74	0.91	0.89	0.93
16	Trauma Scorecard	2607	318	131	1548	610	0.34	0.31	0.37	0.92	0.91	0.93	0.10	0.09	0.13	0.98	0.98	0.98	4.39	3.64	5.30	0.71	0.68	0.75
17	TTR	2607	193	66	1613	735	0.21	0.18	0.23	0.96	0.95	0.97	0.13	0.10	0.16	0.98	0.98	0.98	5.29	4.05	6.92	0.82	0.80	0.85
18	US Field Triage	2607	401	206	1473	527	0.43	0.40	0.46	0.88	0.86	0.89	0.08	0.07	0.10	0.98	0.98	0.99	3.52	3.04	4.08	0.65	0.61	0.69
19	Victoria	2607	559	422	1257	369	0.60	0.57	0.63	0.75	0.73	0.77	0.06	0.05	0.06	0.99	0.99	0.99	2.40	2.17	2.64	0.53	0.49	0.58
20	Vittel	2607	417	211	1468	511	0.45	0.42	0.48	0.87	0.86	0.89	0.08	0.07	0.10	0.98	0.98	0.99	3.58	3.09	4.13	0.63	0.59	0.67
21	WMAS	2607	425	205	1474	503	0.46	0.43	0.49	0.88	0.86	0.89	0.09	0.08	0.10	0.98	0.98	0.99	3.75	3.24	4.34	0.62	0.58	0.66
22	YAS	2607	476	238	1441	452	0.51	0.48	0.55	0.86	0.84	0.87	0.09	0.07	0.10	0.99	0.98	0.99	3.62	3.17	4.14	0.57	0.53	0.61

*Weighted for whole study cohort.

Main analysis findings were not materially changed in sensitivity analyses exploring the influence of study selection criteria and missing data. Including additional primary reference standard positive adult cases involved in acute non-trivial injury events, but where a non-selected working impression code was used, resulted in negligible changes in sensitivity estimates, with specificity remaining unchanged best- and worst-case missing data scenarios, and multiple imputation, resulted in only minor changes in sensitivity/specificity, with the same four triage tools remaining dominant. Further details of sensitivity analysis results are presented in the supplementary materials (S4 Table).

Clear spectrum effects were apparent in triage tool performance across subgroups of different ages or with differing injury characteristics. In elderly patients, adult triage tools demonstrated lower sensitivity and higher specificity for all reference standards. Triage tool accuracy in adults aged between 16 and 64 years demonstrated slightly higher sensitivity and lower specificity across all tools and reference standards compared to the main analysis. Triage tools demonstrated markedly higher sensitivity and lower specificity for the primary MATTS reference standard in penetrating (range: sensitivity = 0.93/specificity = 0.39 to sensitivity = 0.16/specificity = 1.00) compared to blunt injury trauma (range: sensitivity = 0.71/specificity = 0.77 to sensitivity = 0.17/specificity = 0.94). A similar pattern of difference in diagnostic accuracy across triage tools was also apparent in ground level falls (relatively lower sensitivity/higher specificity) compared to higher energy (relatively higher sensitivity/lower specificity) mechanisms of injury. Full details of subgroup analyses are reported in the supplementary materials (S5 Table).

## Discussion

### Summary

Patients presenting to four English trauma networks by ambulance with non-trivial injury were most commonly elderly females with blunt trauma from ground level falls. The overall prevalence of major trauma was low (3.1%). An inverse relationship between specificity and sensitivity for major trauma was observed across studied triage tools, with four optimally performing triage tools identified: Trauma score (sensitivity 0.1, specificity 0.99), MATTS specific tool (sensitivity 0.37, specificity 0.95), MATTS balanced tool (sensitivity 0.58, specificity 0.87), and the MATTS sensitive tool (sensitivity 0.72, specificity 0.76). Spectrum effects were apparent with higher sensitivity and lower specificity in patients aged 16–65, in penetrating trauma, and higher energy mechanisms of injury.

### Interpretation

The theoretical performance of triage tools fell short of recommended ACS-COT targets (sensitivity 95%, specificity 65–75%) [8]. This likely reflects the challenges of assessment of injured patients in the field which suggests that an expectation of high accuracy is unrealistic. Contributory factors could include the heterogeneity of major trauma, lack of point of care tests, limited available information, assessment early in the natural history of the injury, and adverse prehospital environment. Moreover, given the low prevalence of major trauma in injured patients presenting to ambulance services, high specificity is necessary to maintain an acceptable positive predictive value [30]. In fact, economic evaluations of field triage have consistently demonstrated that the ACS-COT targets are not cost-effective, and specificity should be prioritised [10,12].

Together with the Trauma Score, expert consensus developed MATTS triage tools performed the best; with the MATTS specific triage tool (sensitivity 37%, specificity 95%) coming closest to the optimal sensitivity/specificity trade-offs indicated by previous economic evaluations [12]. Overall, the MATTS specific triage tool demonstrated a positive likelihood ratio (LR+) of 7.7 and negative likelihood ratio (LR-) of 0.66. On average, given the observed prevalence of 3.1%, the post-test probability of major trauma would be 20% if the triage tool was positive and 2% if negative. Performance across subgroups varied, with this tool acting as a ‘rule out’ test in penetrating trauma (prevalence 5%, LR+=1.83, LR- = 0.26, post-test probability 9% if positive, 1% if negative); a ‘rule in’ test in elderly patients (pre-test probability 1.9%, LR+=8.04, LR- = 0.77, post-test probability 13% if positive, 1% if negative); and a ‘rule in and rule out’ for high acuity major trauma requiring urgent interventions (pre-test probability 0.9%, LR+=12.33, LR- = 0.38, post-test probability 10% if positive, 0.4% if negative).

It should be noted that the theoretical performance of triage tools was evaluated. This assumed that triage tools were applied to all non-trivially injured patients, were scored according to observed data recorded in ambulance service records, discretionary triage variables (e.g., *consider* bypass to a MTC in patients taking anticoagulants) were not applied, there was full compliance with the result, and no additional clinical acumen was applied. In reality, there is evidence that triage tools are inconsistently applied, final triage decisions often differ from that indicted by the prehospital findings, and unstructured clinical judgement is commonly used [31]. Such practice may improve triage accuracy, generally improving sensitivity at a slight cost to specificity [32]. The MATTS specific triage tool therefore requires prospective evaluation after implementation into practice to gain a clear picture of real-life performance. It could be possible to develop separate triage tools for different subgroups (e.g., high versus low energy injury mechanisms), but multiple options are likely to represent a barrier to use, supporting a more parsimonious single tool approach.

The four included English trauma networks comprised a diverse mix of urbanisation, socioeconomic status, geographies, and injury profiles. Consecutive patients presenting to ambulance services, including helicopter emergency medical services (HEMS), were included. The results of this study should therefore be generalisable throughout the UK National Health Service. The exploration of spectrum effects across different adult ages and injury acuities, should also provide an indication of performance in alternative subgroups. However, external validity to other settings is less certain. Different injury patterns (e.g., higher numbers of gun-shot wounds), alternative health system models (state Vs insurance Vs private funding), and patient demographics (e.g., obesity rates) in other settings could all influence theoretical triage tool performance. Difference in physiology, injury patterns, prevalence, costs, and consequences of paediatric major trauma also prevents extrapolation of results to children. As noted above, real-life triage may differ, and could be affected by differences in prehospital provider training, medicolegal risk, and trauma system organisation (e.g., inclusive versus exclusive design).

### Comparison to literature

Several systematic reviews have examined the theoretical diagnostic accuracy of major trauma triage tools [31,33–35]. These have reported very heterogenous results at high risk of bias, often due to skewed trauma registry study populations, making comparison to the current study difficult. The largest, and most robust, studies to date were performed by Newgard (2016) and Voskens (2017). Newgard et al. evaluated the ACS-COT field triage decision scheme in a sample of 53,487 patients against an ISS ≥ 16 reference standard. Their reported sensitivity of 66.2% and specificity of 87.8% is very similar to those observed herein (ISS ≥ 16 reference standard, sensitivity 70%, specificity 87%) [10]. Voskens and colleagues studied 4,950 injured adults judged by ambulance providers to be high priority (conveyed with flashing lights and siren) [7]. They evaluated the Dutch field triage protocol, demonstrating a sensitivity of 36.2% and specificity of 92.6%. After consideration of spectrum effects from a higher prevalence of major trauma (8.8% vs 3.1%) and younger population (median age 45 Vs 75), the results are consistent with those in from the current study (ISS ≥ 16 reference standard, sensitivity 26%, specificity 93%). Van Rein and colleagues (2019) developed a diagnostic prediction model for major trauma using the same dataset, however external validation is beyond the scope of this paper and requires further study [36].

### Limitations

This study has several strengths. The ‘single-gate’ (‘fishing for all cases in the same pond’) case-cohort design avoids the selection bias inherent in ‘two-gate’ case-control study designs where there is separate selection into the study sample based on reference standard status [37]. Recommendations for data collection in retrospective studies using routine data were followed to minimise information bias [38,39]. Other common sources of bias in diagnostic accuracy studies, including test, diagnostic review, partial verification, incomplete verification, incorporation, and disease progression biases, were avoided [40].

However, there are some potential limitations. The use of routine data may have resulted in index test or reference standard misclassification. Furthermore, reference standard classification is dependent on encompassing TARN inclusion criteria, complete case ascertainment by TARN, and accurate matching of TARN and prehospital data. A detailed critical appraisal of potential biases, classified according to QUADAS-2 criteria, is provided in the supplementary materials (S6 Table) [40].

## Conclusions

Four optimal triage tools, demonstrating a trade-off between sensitivity and specificity, were identified. The choice of ideal tool will depend on the prevalence of major trauma, and relative valuation of false positive and false negative cases; however, previous health economic evaluations would suggest that the MATTS specific tool would be most cost-effective. Further prospective investigation of real-life triage tool performance, including compliance and clinical judgment, is necessary.

## Supporting information

S1 File English NHS Organisation of Major Trauma Services. (DOCX)

S2 Table Expert consensus MATTS specific, sensitive and balanced triage tools. (DOCX)

S3 Table Diagnostic accuracy metrics for selected adult triage tools evaluated against secondary reference standards in patients aged over 16 years. (DOCX)

S4 Table Sensitivity analyses for diagnostic accuracy metrics of selected triage tools evaluated against the primary MATTS reference standard in patients aged over 16 years. (DOCX)

S5 Table Diagnostic accuracy metrics for selected triage tools evaluated against the primary MATTS reference standard in different subgroups. (DOCX)

S6 Table QUADAS-2 Internal and external validity assessment. (DOCX)
