# Supplementary material for: Major Trauma Triage Study (MATTS): Diagnostic accuracy of major trauma triage tools in English regional trauma networks – A case-cohort study
Source: PLoS One. 2026 Mar 27;21(3):e0344996. doi: 10.1371/journal.pone.0344996 (PMC13029787; doi:10.1371/journal.pone.0344996)
Supplement: S1 File — (DOCX) [file pone.0344996.s001.docx]

**Supplementary materials S1**

**English NHS Organisation of Major Trauma Services**

The care of seriously injured patients in England was noted to be sub-optimal in a 2007 report by the National Confidential Enquiry into Patient Outcome and Death, where 60% of cases demonstrated deficiencies in organisational or clinical aspects of care.^18^ A subsequent 2010 National Audit Office enquiry confirmed these findings, highlighting the ad hoc organisation of trauma care, with unacceptable variations in mortality rates, depending on where and when a patient received treatment.^7^

In response, trauma care in England was reconfigured with the introduction of regional trauma networks in 2011, based on the established model of care for injured patients in North America.^19^ Such networks aim to ensure transport of patients injured within a defined geographical area to a hospital matched to their clinical need. These systems of care consist of a central major trauma centre (MTC) hospital providing specialist resuscitation, definitive care and rehabilitation to the highest acuity and most seriously injured patients. Non-specialist general hospitals, termed trauma units (TU), manage lower acuity and less seriously injured patients, and can provide stabilisation and transfer of the more seriously injured to MTCs when needed. Other acute hospitals, which would not routinely manage significantly injured patients, are designated Local Emergency Hospitals (LEH). Management, training, and governance structures are incorporated to coordinate patient management and ensure high quality care is delivered. Trauma networks have subsequently been similarly introduced in Northern Ireland (2017), Scotland (2018) and Wales (2021).

Trauma systems follow one of two broad conceptual models, often referred to as ‘inclusive’ and ‘exclusive’.^20^ The models differ in the extent to which available hospitals can provide major trauma care. In ‘exclusive’ systems, the underpinning philosophy is that all patients with possible major trauma within the area covered by the MTC will be primarily transferred there from the scene, bypassing all other facilities. Exclusive systems are ideally suited for more densely populated urban areas with relatively short pre-hospital journey times and have a lower threshold for transport to the MTC. Conversely, the principle in ‘inclusive’ systems is that, by virtue of geography, longer pre-hospital journey times, MTC capacity constraints, and system efficiency, TUs must be able to receive, stabilise, and subsequently transfer if necessary, appropriate major trauma patients and those not initially requiring critical trauma interventions. Inclusive systems typically have higher thresholds for bypass, may define maximum travel times for transport to MTCs, and could stipulate diversion to the nearest trauma receiving hospital in the contingency of unmanageable airway, ventilation, or haemorrhage. The National Institute for Health and Care Excellence (NICE) major trauma service delivery guidelines recommended implementation of inclusive trauma networks.^21^

Prehospital care for injured patients is complex, varies between regions, and potentially involves overlapping ambulance services, major trauma networks, and specialist services catchment areas (e.g. burns, ortho-plastics). Multiple pre-hospital resources may be available to respond to suspected major trauma cases, including ambulance clinicians, helicopter emergency medical services (HEMS), pre-hospital physician-paramedic teams, or specialist critical care paramedics.^22, 23^ Major trauma specialist services may also be spread across different hospitals, within or across NHS trusts and local healthcare organisations. Moreover, hospital TU and MTC designations may differ depending on paediatric or adult presentations. Remote ambulance service ‘trauma/critical care desks’, offering telephonic advice from senior clinicians, are available in some regions to coordinate the care of patients in the field.

**Major trauma triage**

Only a small fraction of the large number of patients attended by ambulance services following injury represent major trauma, and identification may not always obvious at the scene of incident. It is not feasible to transport all injured patients to MTCs due to limited capacity; many less-severely injured patients can be manged appropriately in TUs and LEHs, and not all patients with major trauma may benefit from MTC care. Ambulance service clinicians therefore require assistance to accurately identify patients that can benefit from expedited MTC care.

In accordance with NICE major trauma service delivery guidelines, NHS ambulance services therefore use pre-hospital triage tools within regional trauma networks.^29^ Their primary purpose is to identify which patients injured within the catchment areas of TUs and LEHs might benefit from prolonged transportation to distant MTCs, bypassing the closer non-specialist hospital. Furthermore, relevant to patients injured in both MTC and non-MTC catchment areas, triage tools have an important secondary role to inform emergency department (ED) pre-alert calls, facilitating patient reception into critical care areas and activation of multi-disciplinary hospital trauma teams to provide rapid, specialised, and coordinated assessment and resuscitation.
