# Supplementary material for: Major Trauma Triage Study (MATTS): Diagnostic accuracy of major trauma triage tools in English regional trauma networks – A case-cohort study
Source: PLoS One. 2026 Mar 27;21(3):e0344996. doi: 10.1371/journal.pone.0344996 (PMC13029787; doi:10.1371/journal.pone.0344996)
Supplement: S2 Table — (DOCX) [file pone.0344996.s002.docx]

**Expert consensus MATTS specific, sensitive and balanced triage tools**

| **Sensitive triage tool** | **Balanced triage tool** | **Specific Triage tool** |
| --- | --- | --- |
| **PHYSIOLOGY**  •Respiratory rate <10, >24  •Shock index >0.9; or SBP <110 in elderly  •New GCS<=14*  **ANATOMICAL INJURIES**  •Chest injury with tachycardia, oxygen requirement, or low oxygen saturations (<94%)  •Chest wall instability, deformity or surgical emphysema  •>=2 long bone fractures  •Open fracture proximal to mid-foot/wrist  •Amputation or mangled extremity proximal to ankle or wrist  •De-gloving injury proximal to mid-foot/wrist  •Penetrating injury proximal to elbows/knees  (i.e. penetrating injury except lower arms and lower legs).  •Suspected open, depressed or CSF leak skull fracture  •Spinal injury with abnormal neurology  •Arterial bleeding requiring control with tourniquet  •Suspected pelvis fracture  **MECHANISM**  •Fall down a full flight of stairs  •Fall >20 feet/2 floors  •Pedestrian/cyclist v train/bus/tram/lorry | **PHYSIOLOGY**  •Sustained respiratory rate <10, >29  •Sustained shock index >1; or SBP <90 (<110 in >65 years)  •Sustained new GCS<=13 (elderly <=14)*  **ANATOMICAL INJURIES**  •Chest injury with tachycardia, oxygen requirement, or low oxygen saturations (<94%)  •>=2 long bone fractures  •Open fracture proximal to mid-foot/wrist  •Amputation or mangled extremity proximal to ankle or wrist  •De-gloving injury proximal to mid-foot/wrist  •Penetrating injury proximal to elbows/knees  •Definite open, depressed or CSF leak skull fracture  •Spinal injury with abnormal neurology  •Arterial bleeding requiring control with tourniquet  •Suspected pelvis fracture | **PHYSIOLOGY**  •Sustained respiratory rate <10 or >29  •Sustained systolic BP<90  •New best GCS motor score ≤4*  **ANATOMICAL INJURIES**  •Chest injury with oxygen requirement / low oxygen saturations (<94%)  •>=2 **proximal** long bone fractures  •Open fracture proximal to mid-foot/wrist  •Amputation or mangled extremity proximal to ankle or wrist  •Penetrating injury to neck, chest, axilla, abdomen, back or groin  • Definite open, depressed or CSF leak skull fracture  •Spinal injury with **paralysis**  •Suspected **major** pelvis fracture |
