# Supplementary material for: Major Trauma Triage Study (MATTS): Diagnostic accuracy of major trauma triage tools in English regional trauma networks – A case-cohort study
Source: PLoS One. 2026 Mar 27;21(3):e0344996. doi: 10.1371/journal.pone.0344996 (PMC13029787; doi:10.1371/journal.pone.0344996)
Supplement: S3 Table — (DOCX) [file pone.0344996.s003.docx]

**SUPPLEMENTARY MATERIALS S3**

**Diagnostic accuracy metrics for selected adult triage tools evaluated against secondary reference standards in patients aged over 16 years.**

| **ISS≥16 reference standard** | | | | | | | | | | | | | | | | | |
| --- | --- | --- | --- | --- | --- | --- | --- | --- | --- | --- | --- | --- | --- | --- | --- | --- | --- |
| **Tool Number** | **Tool Name** | **n** | **Sensitivity** | **LCL** | **UCL** | **Specificity** | **LCL** | **UCL** | **Accuracy** | **TP** | **FP** | **TN** | **FN** | **PPV** | **NPV** | **Positive LR** | **Negative LR** |
| 1 | CRAMS | 2502 | 0.35 | 0.32 | 0.39 | 0.94 | 0.92 | 0.95 | 0.75 | 285 | 110 | 1584 | 523 | 0.11 | 0.99 | 5.43 | 0.69 |
| 2 | Dutch | 2502 | 0.26 | 0.23 | 0.29 | 0.93 | 0.92 | 0.94 | 0.71 | 208 | 117 | 1577 | 600 | 0.08 | 0.98 | 3.73 | 0.80 |
| 3 | Florida | 2502 | 0.41 | 0.37 | 0.44 | 0.86 | 0.84 | 0.87 | 0.71 | 329 | 242 | 1452 | 479 | 0.06 | 0.99 | 2.85 | 0.69 |
| 4 | LAS (current) | 2502 | 0.42 | 0.38 | 0.45 | 0.89 | 0.88 | 0.91 | 0.74 | 338 | 182 | 1512 | 470 | 0.08 | 0.99 | 3.89 | 0.65 |
| 5 | LAS (old) | 2502 | 0.54 | 0.50 | 0.57 | 0.85 | 0.84 | 0.87 | 0.75 | 434 | 246 | 1448 | 374 | 0.08 | 0.99 | 3.70 | 0.54 |
| 6 | MATTS balanced | 2502 | 0.54 | 0.51 | 0.58 | 0.86 | 0.85 | 0.88 | 0.76 | 438 | 229 | 1465 | 370 | 0.08 | 0.99 | 4.01 | 0.53 |
| 7 | MATTS sensitive | 2502 | 0.68 | 0.65 | 0.71 | 0.75 | 0.73 | 0.77 | 0.73 | 548 | 419 | 1275 | 260 | 0.06 | 0.99 | 2.74 | 0.43 |
| 8 | MATTS specific | 2502 | 0.31 | 0.28 | 0.34 | 0.95 | 0.94 | 0.96 | 0.74 | 251 | 88 | 1606 | 557 | 0.12 | 0.98 | 5.98 | 0.73 |
| 9 | MGAP | 2502 | 0.36 | 0.32 | 0.39 | 0.85 | 0.83 | 0.87 | 0.69 | 289 | 253 | 1441 | 519 | 0.05 | 0.98 | 2.40 | 0.76 |
| 10 | North Carolina | 2502 | 0.41 | 0.38 | 0.45 | 0.89 | 0.88 | 0.91 | 0.74 | 333 | 186 | 1508 | 475 | 0.08 | 0.99 | 3.75 | 0.66 |
| 11 | Oregon | 2502 | 0.39 | 0.35 | 0.42 | 0.91 | 0.90 | 0.93 | 0.74 | 312 | 149 | 1545 | 496 | 0.09 | 0.99 | 4.39 | 0.67 |
| 12 | PHI | 2502 | 0.17 | 0.14 | 0.19 | 0.96 | 0.95 | 0.97 | 0.71 | 136 | 66 | 1628 | 672 | 0.09 | 0.98 | 4.32 | 0.87 |
| 13 | RTST | 2502 | 0.34 | 0.30 | 0.37 | 0.93 | 0.92 | 0.94 | 0.74 | 272 | 122 | 1572 | 536 | 0.10 | 0.98 | 4.67 | 0.72 |
| 14 | SWAS | 2502 | 0.29 | 0.26 | 0.32 | 0.95 | 0.94 | 0.96 | 0.74 | 232 | 85 | 1609 | 576 | 0.12 | 0.98 | 5.72 | 0.75 |
| 15 | Trauma Score | 2502 | 0.11 | 0.09 | 0.13 | 0.99 | 0.99 | 1.00 | 0.71 | 88 | 15 | 1679 | 720 | 0.23 | 0.98 | 12.30 | 0.90 |
| 16 | Trauma Scorecard | 2502 | 0.34 | 0.31 | 0.37 | 0.92 | 0.91 | 0.93 | 0.73 | 276 | 133 | 1561 | 532 | 0.09 | 0.98 | 4.35 | 0.72 |
| 17 | TTR | 2502 | 0.21 | 0.18 | 0.24 | 0.96 | 0.95 | 0.97 | 0.72 | 169 | 69 | 1625 | 639 | 0.11 | 0.98 | 5.14 | 0.82 |
| 18 | US Field Triage | 2502 | 0.43 | 0.40 | 0.46 | 0.88 | 0.86 | 0.89 | 0.73 | 347 | 211 | 1483 | 461 | 0.07 | 0.99 | 3.45 | 0.65 |
| 19 | Victoria | 2502 | 0.57 | 0.53 | 0.60 | 0.75 | 0.72 | 0.77 | 0.69 | 457 | 431 | 1263 | 351 | 0.05 | 0.99 | 2.22 | 0.58 |
| 20 | Vittel | 2502 | 0.43 | 0.40 | 0.47 | 0.87 | 0.86 | 0.89 | 0.73 | 349 | 217 | 1477 | 459 | 0.07 | 0.99 | 3.37 | 0.65 |
| 21 | WMAS | 2502 | 0.44 | 0.41 | 0.47 | 0.88 | 0.86 | 0.89 | 0.73 | 355 | 211 | 1483 | 453 | 0.07 | 0.99 | 3.53 | 0.64 |
| 22 | YAS | 2502 | 0.53 | 0.50 | 0.57 | 0.86 | 0.84 | 0.87 | 0.75 | 431 | 243 | 1451 | 377 | 0.08 | 0.99 | 3.72 | 0.55 |
| **MATTS reference standard omitting open fractures** | | | | | | | | | | | | | | | | | |
| **Tool Number** | **Tool Name** | **n** | **Sensitivity** | **LCL** | **UCL** | **Specificity** | **LCL** | **UCL** | **Accuracy** | **TP** | **FP** | **TN** | **FN** | **PPV** | **NPV** | **Positive LR** | **Negative LR** |
| 1 | CRAMS | 2543 | 0.36 | 0.33 | 0.39 | 0.94 | 0.92 | 0.95 | 0.76 | 307 | 108 | 1579 | 549 | 0.12 | 0.98 | 5.60 | 0.69 |
| 2 | Dutch | 2543 | 0.28 | 0.25 | 0.31 | 0.93 | 0.92 | 0.94 | 0.77 | 241 | 114 | 1573 | 615 | 0.09 | 0.98 | 4.17 | 0.77 |
| 3 | Florida | 2543 | 0.42 | 0.39 | 0.46 | 0.86 | 0.84 | 0.87 | 0.75 | 362 | 240 | 1447 | 494 | 0.07 | 0.98 | 2.97 | 0.67 |
| 4 | LAS (current) | 2543 | 0.45 | 0.41 | 0.48 | 0.89 | 0.88 | 0.91 | 0.67 | 383 | 179 | 1508 | 473 | 0.09 | 0.99 | 4.22 | 0.62 |
| 5 | LAS (old) | 2543 | 0.57 | 0.54 | 0.60 | 0.86 | 0.84 | 0.87 | 0.73 | 488 | 242 | 1445 | 368 | 0.09 | 0.99 | 3.97 | 0.50 |
| 6 | MATTS balanced | 2543 | 0.59 | 0.55 | 0.62 | 0.87 | 0.85 | 0.88 | 0.74 | 503 | 223 | 1464 | 353 | 0.10 | 0.99 | 4.45 | 0.48 |
| 7 | MATTS sensitive | 2543 | 0.74 | 0.71 | 0.77 | 0.76 | 0.74 | 0.78 | 0.74 | 631 | 412 | 1275 | 225 | 0.07 | 0.99 | 3.02 | 0.35 |
| 8 | MATTS specific | 2543 | 0.37 | 0.33 | 0.40 | 0.95 | 0.94 | 0.96 | 0.74 | 314 | 84 | 1603 | 542 | 0.15 | 0.98 | 7.37 | 0.67 |
| 9 | MGAP | 2543 | 0.32 | 0.29 | 0.35 | 0.85 | 0.83 | 0.87 | 0.71 | 275 | 255 | 1432 | 581 | 0.04 | 0.98 | 2.13 | 0.80 |
| 10 | North Carolina | 2543 | 0.43 | 0.40 | 0.47 | 0.89 | 0.88 | 0.91 | 0.73 | 370 | 182 | 1505 | 486 | 0.09 | 0.99 | 4.01 | 0.64 |
| 11 | Oregon | 2543 | 0.40 | 0.36 | 0.43 | 0.91 | 0.90 | 0.93 | 0.71 | 340 | 146 | 1541 | 516 | 0.10 | 0.99 | 4.59 | 0.66 |
| 12 | PHI | 2543 | 0.19 | 0.16 | 0.22 | 0.96 | 0.95 | 0.97 | 0.75 | 163 | 66 | 1621 | 693 | 0.10 | 0.98 | 4.87 | 0.84 |
| 13 | RTST | 2543 | 0.33 | 0.30 | 0.36 | 0.93 | 0.92 | 0.94 | 0.75 | 281 | 121 | 1566 | 575 | 0.10 | 0.98 | 4.58 | 0.72 |
| 14 | SWAS | 2543 | 0.31 | 0.28 | 0.34 | 0.95 | 0.94 | 0.96 | 0.74 | 267 | 83 | 1604 | 589 | 0.13 | 0.98 | 6.34 | 0.72 |
| 15 | Trauma Score | 2543 | 0.11 | 0.09 | 0.13 | 0.99 | 0.99 | 1.00 | 0.74 | 92 | 14 | 1673 | 764 | 0.26 | 0.98 | 12.95 | 0.90 |
| 16 | Trauma Scorecard | 2543 | 0.36 | 0.33 | 0.39 | 0.92 | 0.91 | 0.94 | 0.74 | 306 | 131 | 1556 | 550 | 0.10 | 0.98 | 4.60 | 0.70 |
| 17 | TTR | 2543 | 0.22 | 0.19 | 0.25 | 0.96 | 0.95 | 0.97 | 0.74 | 188 | 66 | 1621 | 668 | 0.12 | 0.98 | 5.61 | 0.81 |
| 18 | US Field Triage | 2543 | 0.45 | 0.42 | 0.48 | 0.88 | 0.86 | 0.89 | 0.70 | 386 | 207 | 1480 | 470 | 0.08 | 0.99 | 3.68 | 0.63 |
| 19 | Victoria | 2543 | 0.60 | 0.56 | 0.63 | 0.75 | 0.73 | 0.77 | 0.69 | 511 | 426 | 1261 | 345 | 0.05 | 0.99 | 2.36 | 0.54 |
| 20 | Vittel | 2543 | 0.46 | 0.43 | 0.49 | 0.87 | 0.86 | 0.89 | 0.73 | 394 | 212 | 1475 | 462 | 0.08 | 0.99 | 3.66 | 0.62 |
| 21 | WMAS | 2543 | 0.47 | 0.44 | 0.51 | 0.88 | 0.86 | 0.89 | 0.70 | 404 | 207 | 1480 | 452 | 0.08 | 0.99 | 3.85 | 0.60 |
| 22 | YAS | 2543 | 0.55 | 0.52 | 0.58 | 0.86 | 0.84 | 0.87 | 0.73 | 470 | 239 | 1448 | 386 | 0.08 | 0.99 | 3.88 | 0.53 |
| **Urgent interventions reference standard** | | | | | | | | | | | | | | | | | |
| **Tool Number** | **Tool Name** | **n** | **Sensitivity** | **LCL** | **UCL** | **Specificity** | **LCL** | **UCL** | **Accuracy** | **TP** | **FP** | **TN** | **FN** | **PPV** | **NPV** | **Positive LR** | **Negative LR** |
| 1 | CRAMS | 2003 | 0.55 | 0.50 | 0.61 | 0.93 | 0.92 | 0.95 | 0.88 | 158 | 113 | 1605 | 127 | 0.06 | 1.00 | 8.43 | 0.48 |
| 2 | Dutch | 2003 | 0.55 | 0.49 | 0.61 | 0.93 | 0.92 | 0.94 | 0.88 | 157 | 119 | 1599 | 128 | 0.06 | 1.00 | 7.95 | 0.48 |
| 3 | Florida | 2003 | 0.71 | 0.65 | 0.76 | 0.86 | 0.84 | 0.87 | 0.84 | 201 | 245 | 1473 | 84 | 0.04 | 1.00 | 4.95 | 0.34 |
| 4 | LAS (current) | 2003 | 0.71 | 0.66 | 0.76 | 0.89 | 0.88 | 0.91 | 0.87 | 202 | 184 | 1534 | 83 | 0.05 | 1.00 | 6.62 | 0.33 |
| 5 | LAS (old) | 2003 | 0.77 | 0.72 | 0.82 | 0.85 | 0.84 | 0.87 | 0.84 | 220 | 254 | 1464 | 65 | 0.04 | 1.00 | 5.22 | 0.27 |
| 6 | MATTS balanced | 2003 | 0.79 | 0.74 | 0.84 | 0.86 | 0.85 | 0.88 | 0.85 | 225 | 234 | 1484 | 60 | 0.04 | 1.00 | 5.80 | 0.24 |
| 7 | MATTS sensitive | 2003 | 0.90 | 0.86 | 0.93 | 0.75 | 0.73 | 0.77 | 0.77 | 256 | 431 | 1287 | 29 | 0.03 | 1.00 | 3.58 | 0.14 |
| 8 | MATTS specific | 2003 | 0.64 | 0.58 | 0.69 | 0.95 | 0.94 | 0.96 | 0.90 | 182 | 89 | 1629 | 103 | 0.09 | 1.00 | 12.33 | 0.38 |
| 9 | MGAP | 2003 | 0.44 | 0.38 | 0.50 | 0.85 | 0.83 | 0.87 | 0.79 | 125 | 259 | 1459 | 160 | 0.02 | 0.99 | 2.91 | 0.66 |
| 10 | North Carolina | 2003 | 0.70 | 0.64 | 0.75 | 0.89 | 0.87 | 0.90 | 0.86 | 199 | 190 | 1528 | 86 | 0.05 | 1.00 | 6.31 | 0.34 |
| 11 | Oregon | 2003 | 0.65 | 0.59 | 0.70 | 0.91 | 0.90 | 0.93 | 0.87 | 184 | 151 | 1567 | 101 | 0.06 | 1.00 | 7.35 | 0.39 |
| 12 | PHI | 2003 | 0.40 | 0.34 | 0.46 | 0.96 | 0.95 | 0.97 | 0.88 | 114 | 67 | 1651 | 171 | 0.07 | 1.00 | 10.26 | 0.62 |
| 13 | RTST | 2003 | 0.53 | 0.47 | 0.59 | 0.93 | 0.91 | 0.94 | 0.87 | 151 | 125 | 1593 | 134 | 0.05 | 1.00 | 7.28 | 0.51 |
| 14 | SWAS | 2003 | 0.58 | 0.53 | 0.64 | 0.95 | 0.94 | 0.96 | 0.90 | 166 | 90 | 1628 | 119 | 0.08 | 1.00 | 11.12 | 0.44 |
| 15 | Trauma Score | 2003 | 0.27 | 0.22 | 0.33 | 0.99 | 0.99 | 1.00 | 0.89 | 78 | 14 | 1704 | 207 | 0.23 | 0.99 | 33.59 | 0.73 |
| 16 | Trauma Scorecard | 2003 | 0.61 | 0.55 | 0.66 | 0.92 | 0.91 | 0.93 | 0.88 | 173 | 136 | 1582 | 112 | 0.06 | 1.00 | 7.67 | 0.43 |
| 17 | TTR | 2003 | 0.47 | 0.41 | 0.53 | 0.96 | 0.95 | 0.97 | 0.89 | 134 | 68 | 1650 | 151 | 0.09 | 1.00 | 11.88 | 0.55 |
| 18 | US Field Triage | 2003 | 0.70 | 0.65 | 0.75 | 0.87 | 0.86 | 0.89 | 0.85 | 200 | 216 | 1502 | 85 | 0.04 | 1.00 | 5.58 | 0.34 |
| 19 | Victoria | 2003 | 0.82 | 0.77 | 0.86 | 0.74 | 0.72 | 0.76 | 0.75 | 233 | 441 | 1277 | 52 | 0.02 | 1.00 | 3.19 | 0.25 |
| 20 | Vittel | 2003 | 0.73 | 0.68 | 0.78 | 0.87 | 0.85 | 0.89 | 0.85 | 208 | 223 | 1495 | 77 | 0.04 | 1.00 | 5.62 | 0.31 |
| 21 | WMAS | 2003 | 0.74 | 0.69 | 0.79 | 0.87 | 0.86 | 0.89 | 0.85 | 210 | 217 | 1501 | 75 | 0.04 | 1.00 | 5.83 | 0.30 |
| 22 | YAS | 2003 | 0.72 | 0.67 | 0.77 | 0.85 | 0.84 | 0.87 | 0.84 | 206 | 251 | 1467 | 79 | 0.04 | 1.00 | 4.95 | 0.33 |

CRAMS: Circulation, Respiration, Abdomen, Motor, and Speech Scale; Dutch: Dutch Field Triage Protocol; Florida: State of Florida Trauma Criteria; LAS (current): London Ambulance Service (current) Major Trauma Triage Tool; LAS (old): London Ambulance Service (old) Major Trauma Triage Tool; MATTS balanced: Newly developed MATTS triage tool – balancing sensitivity/specificity; MATTS sensitive: Newly developed MATTS triage tool – prioritising sensitivity; MATTS specific: Newly developed MATTS triage tool – prioritising specificity; MGAP: Mechanism, Glasgow Coma Scale, Age, and Arterial Pressure Score; North Carolina: North Carolina Trauma and Burn EMS Triage and Destination Plan; Oregon: Oregon Guidelines for Field Triage of Injured Patients; PHI: The Prehospital Index; RTST: Triage Revised Trauma Score; SWAS: South West Ambulance Service Major Trauma Triage Tool; Trauma Score: Trauma Score; Trauma Scorecard: Trauma Scorecard; TTR: Trauma Triage Rule; US Field Triage: National Guidelines for the Field Triage of Injured Patients (2011); Victoria: Pre-hospital Major Trauma Triage - Trauma Victoria; Vittel: Vittel criteria for severe trauma triage; WMAS: West Midlands Ambulance Service Major Trauma Triage Tool: YAS Yorkshire Ambulance Service Major Trauma Triage Tool
