# Supplementary material for: Major Trauma Triage Study (MATTS): Diagnostic accuracy of major trauma triage tools in English regional trauma networks – A case-cohort study
Source: PLoS One. 2026 Mar 27;21(3):e0344996. doi: 10.1371/journal.pone.0344996 (PMC13029787; doi:10.1371/journal.pone.0344996)
Supplement: S4 Table — (DOCX) [file pone.0344996.s004.docx]

**SUPPLEMENTARY MATERIALS S4**

**Sensitivity analyses for diagnostic accuracy metrics of selected triage tools evaluated against the primary MATTS reference standard in patients aged over 16 years**

| **Including reference standard positive cases with non-selected working impression codes.** | | | | | | | | | | | | | | | | | | | | | | | | | | | | | | | | | | | | | | | | | | | | | | | | | | | | | | | | | | | | | | | | | |  |  |  |
| --- | --- | --- | --- | --- | --- | --- | --- | --- | --- | --- | --- | --- | --- | --- | --- | --- | --- | --- | --- | --- | --- | --- | --- | --- | --- | --- | --- | --- | --- | --- | --- | --- | --- | --- | --- | --- | --- | --- | --- | --- | --- | --- | --- | --- | --- | --- | --- | --- | --- | --- | --- | --- | --- | --- | --- | --- | --- | --- | --- | --- | --- | --- | --- | --- | --- | --- | --- | --- |
| Tool no. | | Tool name | | | n | TP | | | FP | | TN | | | | FN | | | Sensitivity | | | Sens LCL | | | | Sens UCL | | | Specificity | | | Spec LCL | | | Spec UCL | | | PPV | | | | NPV | | | Positive LR | | | Positive LR LCL | | | | | Positive LR UCL | | | | Negative LR | | Negative LR LCL | | | Negative LR UCL | | | | |  |  |  |
| 1 | | CRAMS | | | 2,680 | 327 | | | 107 | | 1,572 | | | | 674 | | | 0.33 | | | 0.30 | | | | 0.36 | | | 0.94 | | | 0.93 | | | 0.95 | | | 0.12 | | | | 0.98 | | | 5.50 | | | 4.49 | | | | | 6.74 | | | | 0.71 | | 0.59 | | | 0.86 | | | | |  |  |  |
| 2 | | Dutch | | | 2,680 | 261 | | | 113 | | 1,566 | | | | 740 | | | 0.26 | | | 0.23 | | | | 0.29 | | | 0.93 | | | 0.92 | | | 0.94 | | | 0.09 | | | | 0.98 | | | 3.71 | | | 3.02 | | | | | 4.57 | | | | 0.80 | | 0.66 | | | 0.95 | | | | |  |  |  |
| 3 | | Florida | | | 2,680 | 392 | | | 239 | | 1,440 | | | | 609 | | | 0.39 | | | 0.36 | | | | 0.42 | | | 0.86 | | | 0.84 | | | 0.88 | | | 0.07 | | | | 0.98 | | | 2.79 | | | 2.42 | | | | | 3.21 | | | | 0.71 | | 0.62 | | | 0.81 | | | | |  |  |  |
| 4 | | LAS (current) | | | 2,680 | 436 | | | 176 | | 1,503 | | | | 565 | | | 0.44 | | | 0.41 | | | | 0.47 | | | 0.90 | | | 0.89 | | | 0.91 | | | 0.10 | | | | 0.98 | | | 4.40 | | | 3.76 | | | | | 5.15 | | | | 0.62 | | 0.54 | | | 0.72 | | | | |  |  |  |
| 5 | | LAS (old) | | | 2,680 | 553 | | | 238 | | 1,441 | | | | 448 | | | 0.55 | | | 0.52 | | | | 0.58 | | | 0.86 | | | 0.84 | | | 0.88 | | | 0.09 | | | | 0.99 | | | 3.93 | | | 3.45 | | | | | 4.48 | | | | 0.52 | | 0.46 | | | 0.60 | | | | |  |  |  |
| 6 | | MATTS balanced | | | 2,680 | 571 | | | 219 | | 1,460 | | | | 430 | | | 0.57 | | | 0.54 | | | | 0.60 | | | 0.87 | | | 0.85 | | | 0.89 | | | 0.10 | | | | 0.99 | | | 4.38 | | | 3.83 | | | | | 5.02 | | | | 0.49 | | 0.43 | | | 0.57 | | | | |  |  |  |
| 7 | | MATTS sensitive | | | 2,680 | 716 | | | 407 | | 1,272 | | | | 285 | | | 0.72 | | | 0.69 | | | | 0.75 | | | 0.76 | | | 0.74 | | | 0.78 | | | 0.07 | | | | 0.99 | | | 3.00 | | | 2.73 | | | | | 3.29 | | | | 0.37 | | 0.32 | | | 0.42 | | | | |  |  |  |
| 8 | | MATTS specific | | | 2,680 | 369 | | | 81 | | 1,598 | | | | 632 | | | 0.37 | | | 0.34 | | | | 0.40 | | | 0.95 | | | 0.94 | | | 0.96 | | | 0.17 | | | | 0.98 | | | 7.40 | | | 5.89 | | | | | 9.29 | | | | 0.66 | | 0.53 | | | 0.82 | | | | |  |  |  |
| 9 | | MGAP | | | 2,680 | 305 | | | 253 | | 1,426 | | | | 696 | | | 0.30 | | | 0.27 | | | | 0.33 | | | 0.85 | | | 0.83 | | | 0.87 | | | 0.05 | | | | 0.98 | | | 2.00 | | | 1.73 | | | | | 2.32 | | | | 0.82 | | 0.73 | | | 0.93 | | | | |  |  |  |
| 10 | | North Carolina | | | 2,680 | 405 | | | 181 | | 1,498 | | | | 596 | | | 0.40 | | | 0.37 | | | | 0.43 | | | 0.89 | | | 0.88 | | | 0.90 | | | 0.09 | | | | 0.98 | | | 3.64 | | | 3.11 | | | | | 4.25 | | | | 0.67 | | 0.58 | | | 0.78 | | | | |  |  |  |
| 11 | | Oregon | | | 2,680 | 366 | | | 146 | | 1,533 | | | | 635 | | | 0.37 | | | 0.34 | | | | 0.40 | | | 0.91 | | | 0.90 | | | 0.92 | | | 0.10 | | | | 0.98 | | | 4.11 | | | 3.45 | | | | | 4.90 | | | | 0.69 | | 0.59 | | | 0.81 | | | | |  |  |  |
| 12 | | PHI | | | 2,680 | 171 | | | 66 | | 1,613 | | | | 830 | | | 0.17 | | | 0.15 | | | | 0.19 | | | 0.96 | | | 0.95 | | | 0.97 | | | 0.11 | | | | 0.98 | | | 4.25 | | | 3.23 | | | | | 5.58 | | | | 0.86 | | 0.68 | | | 1.10 | | | | |  |  |  |
| 13 | | RTST | | | 2,680 | 302 | | | 121 | | 1,558 | | | | 699 | | | 0.30 | | | 0.27 | | | | 0.33 | | | 0.93 | | | 0.92 | | | 0.94 | | | 0.10 | | | | 0.98 | | | 4.29 | | | 3.52 | | | | | 5.21 | | | | 0.75 | | 0.63 | | | 0.90 | | | | |  |  |  |
| 14 | | SWAS | | | 2,680 | 319 | | | 80 | | 1,599 | | | | 682 | | | 0.32 | | | 0.29 | | | | 0.35 | | | 0.95 | | | 0.94 | | | 0.96 | | | 0.15 | | | | 0.98 | | | 6.40 | | | 5.07 | | | | | 8.07 | | | | 0.72 | | 0.58 | | | 0.89 | | | | |  |  |  |
| 15 | | Trauma Score | | | 2,680 | 96 | | | 14 | | 1,665 | | | | 905 | | | 0.10 | | | 0.08 | | | | 0.12 | | | 0.99 | | | 0.99 | | | 0.99 | | | 0.26 | | | | 0.98 | | | 10.00 | | | 5.74 | | | | | 17.42 | | | | 0.91 | | 0.54 | | | 1.53 | | | | |  |  |  |
| 16 | | Trauma Scorecard | | | 2,680 | 333 | | | 131 | | 1,548 | | | | 668 | | | 0.33 | | | 0.30 | | | | 0.36 | | | 0.92 | | | 0.91 | | | 0.93 | | | 0.10 | | | | 0.98 | | | 4.13 | | | 3.42 | | | | | 4.97 | | | | 0.73 | | 0.61 | | | 0.86 | | | | |  |  |  |
| 17 | | TTR | | | 2,680 | 199 | | | 66 | | 1,613 | | | | 802 | | | 0.20 | | | 0.18 | | | | 0.22 | | | 0.96 | | | 0.95 | | | 0.97 | | | 0.13 | | | | 0.98 | | | 5.00 | | | 3.83 | | | | | 6.53 | | | | 0.83 | | 0.66 | | | 1.06 | | | | |  |  |  |
| 18 | | US Field Triage | | | 2,680 | 422 | | | 206 | | 1,473 | | | | 579 | | | 0.42 | | | 0.39 | | | | 0.45 | | | 0.88 | | | 0.86 | | | 0.90 | | | 0.08 | | | | 0.98 | | | 3.50 | | | 3.02 | | | | | 4.05 | | | | 0.66 | | 0.57 | | | 0.76 | | | | |  |  |  |
| 19 | | Victoria | | | 2,680 | 588 | | | 422 | | 1,257 | | | | 413 | | | 0.59 | | | 0.56 | | | | 0.62 | | | 0.75 | | | 0.73 | | | 0.77 | | | 0.06 | | | | 0.99 | | | 2.36 | | | 2.14 | | | | | 2.60 | | | | 0.55 | | 0.49 | | | 0.61 | | | | |  |  |  |
| 20 | | Vittel | | | 2,680 | 437 | | | 211 | | 1,468 | | | | 564 | | | 0.44 | | | 0.41 | | | | 0.47 | | | 0.87 | | | 0.85 | | | 0.89 | | | 0.08 | | | | 0.98 | | | 3.38 | | | 2.93 | | | | | 3.91 | | | | 0.64 | | 0.56 | | | 0.74 | | | | |  |  |  |
| 21 | | WMAS | | | 2,680 | 447 | | | 205 | | 1,474 | | | | 554 | | | 0.45 | | | 0.42 | | | | 0.48 | | | 0.88 | | | 0.86 | | | 0.90 | | | 0.09 | | | | 0.98 | | | 3.75 | | | 3.24 | | | | | 4.34 | | | | 0.62 | | 0.54 | | | 0.72 | | | | |  |  |  |
| 22 | | YAS | | | 2,680 | 510 | | | 238 | | 1,441 | | | | 491 | | | 0.51 | | | 0.48 | | | | 0.54 | | | 0.86 | | | 0.84 | | | 0.88 | | | 0.09 | | | | 0.99 | | | 3.64 | | | 3.19 | | | | | 4.16 | | | | 0.57 | | 0.50 | | | 0.65 | | | | |  |  |  |
| **BEST CASE MISSING DATA SCENARIO: Missing data assumed to be triage tool negative** | | | | | | | | | | | | | | | | | | | | | | | | | | | | | | | | | | | | | | | | | | | | | | | | | | | | | | | | | | | | | | | | | | | |  |
| Tool number | | | Tool name | | | | n | | | TP | | | FP | | | TN | | | FN | | | Sensitivity | | | | Sens LCL | | | Sens UCL | | Specificity | | | | Spec LCL | | | Spec UCL | | | | PPV | | | NPV | | | Positive LR | | | Positive LR LCL | | | Positive  LR  UCL | | | | | Negative LR | | | Negative LR LCL | | | Negative LR UCL | | |  |
| 1 | | | CRAMS | | | | 2,790 | | | 343 | | | 115 | | | 1,666 | | | 666 | | | 0.34 | | | | 0.31 | | | 0.37 | | 0.94 | | | | 0.92 | | | 0.95 | | | | 0.12 | | | 0.98 | | | 5.26 | | | 4.33 | | | 6.41 | | | | | 0.71 | | | 0.59 | | | 0.85 | | |  |
| 2 | | | Dutch | | | | 2,790 | | | 293 | | | 124 | | | 1,657 | | | 716 | | | 0.29 | | | | 0.26 | | | 0.32 | | 0.93 | | | | 0.92 | | | 0.94 | | | | 0.10 | | | 0.98 | | | 4.17 | | | 3.43 | | | 5.07 | | | | | 0.76 | | | 0.64 | | | 0.91 | | |  |
| 3 | | | Florida | | | | 2,790 | | | 429 | | | 253 | | | 1,528 | | | 580 | | | 0.43 | | | | 0.39 | | | 0.46 | | 0.86 | | | | 0.84 | | | 0.87 | | | | 0.07 | | | 0.98 | | | 2.99 | | | 2.62 | | | 3.42 | | | | | 0.67 | | | 0.59 | | | 0.76 | | |  |
| 4 | | | LAS (current) | | | | 2,790 | | | 460 | | | 189 | | | 1,592 | | | 549 | | | 0.46 | | | | 0.43 | | | 0.49 | | 0.89 | | | | 0.88 | | | 0.91 | | | | 0.11 | | | 0.98 | | | 4.30 | | | 3.70 | | | 4.99 | | | | | 0.61 | | | 0.53 | | | 0.70 | | |  |
| 5 | | | LAS (old) | | | | 2,790 | | | 569 | | | 251 | | | 1,530 | | | 440 | | | 0.56 | | | | 0.53 | | | 0.59 | | 0.86 | | | | 0.84 | | | 0.88 | | | | 0.10 | | | 0.99 | | | 4.00 | | | 3.52 | | | 4.54 | | | | | 0.51 | | | 0.44 | | | 0.58 | | |  |
| 6 | | | MATTS balanced | | | | 2,790 | | | 588 | | | 236 | | | 1,545 | | | 421 | | | 0.58 | | | | 0.55 | | | 0.61 | | 0.87 | | | | 0.85 | | | 0.88 | | | | 0.11 | | | 0.99 | | | 4.40 | | | 3.86 | | | 5.01 | | | | | 0.48 | | | 0.42 | | | 0.55 | | |  |
| 7 | | | MATTS sensitive | | | | 2,790 | | | 732 | | | 435 | | | 1,346 | | | 277 | | | 0.73 | | | | 0.70 | | | 0.75 | | 0.76 | | | | 0.74 | | | 0.78 | | | | 0.07 | | | 0.99 | | | 2.97 | | | 2.71 | | | 3.25 | | | | | 0.36 | | | 0.32 | | | 0.41 | | |  |
| 8 | | | MATTS specific | | | | 2,790 | | | 385 | | | 87 | | | 1,694 | | | 624 | | | 0.38 | | | | 0.35 | | | 0.41 | | 0.95 | | | | 0.94 | | | 0.96 | | | | 0.18 | | | 0.98 | | | 7.81 | | | 6.27 | | | 9.73 | | | | | 0.65 | | | 0.53 | | | 0.80 | | |  |
| 9 | | | MGAP | | | | 2,790 | | | 316 | | | 271 | | | 1,510 | | | 693 | | | 0.31 | | | | 0.28 | | | 0.34 | | 0.85 | | | | 0.83 | | | 0.86 | | | | 0.05 | | | 0.98 | | | 2.06 | | | 1.78 | | | 2.37 | | | | | 0.81 | | | 0.72 | | | 0.91 | | |  |
| 10 | | | North Carolina | | | | 2,790 | | | 434 | | | 190 | | | 1,591 | | | 575 | | | 0.43 | | | | 0.40 | | | 0.46 | | 0.89 | | | | 0.88 | | | 0.91 | | | | 0.10 | | | 0.98 | | | 4.03 | | | 3.46 | | | 4.69 | | | | | 0.64 | | | 0.55 | | | 0.74 | | |  |
| 11 | | | Oregon | | | | 2,790 | | | 396 | | | 154 | | | 1,627 | | | 613 | | | 0.39 | | | | 0.36 | | | 0.42 | | 0.91 | | | | 0.90 | | | 0.93 | | | | 0.11 | | | 0.98 | | | 4.54 | | | 3.83 | | | 5.38 | | | | | 0.67 | | | 0.57 | | | 0.78 | | |  |
| 12 | | | PHI | | | | 2,790 | | | 202 | | | 71 | | | 1,710 | | | 807 | | | 0.20 | | | | 0.18 | | | 0.22 | | 0.96 | | | | 0.95 | | | 0.97 | | | | 0.12 | | | 0.98 | | | 5.02 | | | 3.88 | | | 6.51 | | | | | 0.83 | | | 0.66 | | | 1.05 | | |  |
| 13 | | | RTST | | | | 2,790 | | | 326 | | | 128 | | | 1,653 | | | 683 | | | 0.32 | | | | 0.29 | | | 0.35 | | 0.93 | | | | 0.92 | | | 0.94 | | | | 0.11 | | | 0.98 | | | 4.50 | | | 3.72 | | | 5.43 | | | | | 0.73 | | | 0.61 | | | 0.87 | | |  |
| 14 | | | SWAS | | | | 2,790 | | | 337 | | | 88 | | | 1,693 | | | 672 | | | 0.33 | | | | 0.30 | | | 0.36 | | 0.95 | | | | 0.94 | | | 0.96 | | | | 0.15 | | | 0.98 | | | 6.76 | | | 5.42 | | | 8.44 | | | | | 0.70 | | | 0.57 | | | 0.86 | | |  |
| 15 | | | Trauma Score | | | | 2,790 | | | 106 | | | 16 | | | 1,765 | | | 903 | | | 0.11 | | | | 0.09 | | | 0.12 | | 0.99 | | | | 0.99 | | | 1.00 | | | | 0.26 | | | 0.98 | | | 11.69 | | | 6.95 | | | 19.67 | | | | | 0.90 | | | 0.55 | | | 1.47 | | |  |
| 16 | | | Trauma Scorecard | | | | 2,790 | | | 361 | | | 139 | | | 1,642 | | | 648 | | | 0.36 | | | | 0.33 | | | 0.39 | | 0.92 | | | | 0.91 | | | 0.93 | | | | 0.11 | | | 0.98 | | | 4.58 | | | 3.83 | | | 5.49 | | | | | 0.70 | | | 0.59 | | | 0.82 | | |  |
| 17 | | | TTR | | | | 2,790 | | | 222 | | | 71 | | | 1,710 | | | 787 | | | 0.22 | | | | 0.19 | | | 0.25 | | 0.96 | | | | 0.95 | | | 0.97 | | | | 0.13 | | | 0.98 | | | 5.52 | | | 4.27 | | | 7.13 | | | | | 0.81 | | | 0.65 | | | 1.02 | | |  |
| 18 | | | US Field Triage | | | | 2,790 | | | 450 | | | 219 | | | 1,562 | | | 559 | | | 0.45 | | | | 0.42 | | | 0.48 | | 0.88 | | | | 0.86 | | | 0.89 | | | | 0.09 | | | 0.98 | | | 3.63 | | | 3.15 | | | 4.18 | | | | | 0.63 | | | 0.55 | | | 0.72 | | |  |
| 19 | | | Victoria | | | | 2,790 | | | 620 | | | 447 | | | 1,334 | | | 389 | | | 0.61 | | | | 0.58 | | | 0.64 | | 0.75 | | | | 0.73 | | | 0.77 | | | | 0.06 | | | 0.99 | | | 2.45 | | | 2.23 | | | 2.69 | | | | | 0.51 | | | 0.46 | | | 0.58 | | |  |
| 20 | | | Vittel | | | | 2,790 | | | 475 | | | 226 | | | 1,555 | | | 534 | | | 0.47 | | | | 0.44 | | | 0.50 | | 0.87 | | | | 0.86 | | | 0.89 | | | | 0.09 | | | 0.98 | | | 3.71 | | | 3.23 | | | 4.26 | | | | | 0.61 | | | 0.53 | | | 0.69 | | |  |
| 21 | | | WMAS | | | | 2,790 | | | 478 | | | 219 | | | 1,562 | | | 531 | | | 0.47 | | | | 0.44 | | | 0.50 | | 0.88 | | | | 0.86 | | | 0.89 | | | | 0.09 | | | 0.98 | | | 3.85 | | | 3.35 | | | 4.43 | | | | | 0.60 | | | 0.52 | | | 0.69 | | |  |
| 22 | | | YAS | | | | 2,790 | | | 524 | | | 251 | | | 1,530 | | | 485 | | | 0.52 | | | | 0.49 | | | 0.55 | | 0.86 | | | | 0.84 | | | 0.88 | | | | 0.09 | | | 0.99 | | | 3.68 | | | 3.24 | | | 4.19 | | | | | 0.56 | | | 0.49 | | | 0.64 | | |  |
| **WORST CASE MISSING DATA SCENARIO: Missing data assumed to be triage tool positive** | | | | | | | | | | | | | | | | | | | | | | | | | | | | | | | | | | | | | | | | | | | | | | | | | | | | | | | | | | | | | | | | | | | | |
| Tool number | Tool name | | | | | | | n | | | | TP | | FP | | | TN | | | FN | | | Sensitivity | | | | Sens LCL | | | Sens UCL | | Specificity | | | | Spec LCL | | | Spec UCL | | | | PPV | | | NPV | | | Positive LR | | | | Positive LR LCL | | Positive  LR  UCL | | | | | Negative LR | | | Negative LR LCL | | | | Negative LR UCL | |
| 1 | CRAMS | | | | | | | 2,790 | | | | 380 | | 176 | | | 1,605 | | | 629 | | | 0.38 | | | | 0.35 | | | 0.41 | | 0.90 | | | | 0.89 | | | 0.92 | | | | 0.09 | | | 0.98 | | | 3.81 | | | | 3.24 | | 4.48 | | | | | 0.69 | | | 0.60 | | | | 0.80 | |
| 2 | Dutch | | | | | | | 2,790 | | | | 322 | | 182 | | | 1,599 | | | 687 | | | 0.32 | | | | 0.29 | | | 0.35 | | 0.90 | | | | 0.88 | | | 0.91 | | | | 0.08 | | | 0.98 | | | 3.12 | | | | 2.65 | | 3.68 | | | | | 0.76 | | | 0.66 | | | | 0.88 | |
| 3 | Florida | | | | | | | 2,790 | | | | 456 | | 326 | | | 1,455 | | | 553 | | | 0.45 | | | | 0.42 | | | 0.48 | | 0.82 | | | | 0.80 | | | 0.83 | | | | 0.06 | | | 0.98 | | | 2.47 | | | | 2.19 | | 2.78 | | | | | 0.67 | | | 0.60 | | | | 0.75 | |
| 4 | LAS (current) | | | | | | | 2,790 | | | | 495 | | 255 | | | 1,526 | | | 514 | | | 0.49 | | | | 0.46 | | | 0.52 | | 0.86 | | | | 0.84 | | | 0.87 | | | | 0.08 | | | 0.98 | | | 3.43 | | | | 3.01 | | 3.90 | | | | | 0.59 | | | 0.52 | | | | 0.68 | |
| 5 | LAS (old) | | | | | | | 2,790 | | | | 600 | | 318 | | | 1,463 | | | 409 | | | 0.59 | | | | 0.56 | | | 0.62 | | 0.82 | | | | 0.80 | | | 0.84 | | | | 0.08 | | | 0.99 | | | 3.33 | | | | 2.98 | | 3.72 | | | | | 0.49 | | | 0.44 | | | | 0.56 | |
| 6 | MATTS balanced | | | | | | | 2,790 | | | | 620 | | 297 | | | 1,484 | | | 389 | | | 0.61 | | | | 0.58 | | | 0.64 | | 0.83 | | | | 0.82 | | | 0.85 | | | | 0.09 | | | 0.99 | | | 3.68 | | | | 3.29 | | 4.13 | | | | | 0.46 | | | 0.41 | | | | 0.53 | |
| 7 | MATTS sensitive | | | | | | | 2,790 | | | | 754 | | 490 | | | 1,291 | | | 255 | | | 0.75 | | | | 0.72 | | | 0.77 | | 0.72 | | | | 0.70 | | | 0.75 | | | | 0.07 | | | 0.99 | | | 2.72 | | | | 2.50 | | 2.95 | | | | | 0.35 | | | 0.31 | | | | 0.40 | |
| 8 | MATTS specific | | | | | | | 2,790 | | | | 444 | | 155 | | | 1,626 | | | 565 | | | 0.44 | | | | 0.41 | | | 0.47 | | 0.91 | | | | 0.90 | | | 0.93 | | | | 0.12 | | | 0.98 | | | 5.06 | | | | 4.28 | | 5.97 | | | | | 0.61 | | | 0.52 | | | | 0.72 | |
| 9 | MGAP | | | | | | | 2,790 | | | | 358 | | 312 | | | 1,469 | | | 651 | | | 0.35 | | | | 0.33 | | | 0.38 | | 0.82 | | | | 0.81 | | | 0.84 | | | | 0.05 | | | 0.98 | | | 2.03 | | | | 1.78 | | 2.31 | | | | | 0.78 | | | 0.70 | | | | 0.87 | |
| 10 | North Carolina | | | | | | | 2,790 | | | | 464 | | 261 | | | 1,520 | | | 545 | | | 0.46 | | | | 0.43 | | | 0.49 | | 0.85 | | | | 0.84 | | | 0.87 | | | | 0.08 | | | 0.98 | | | 3.14 | | | | 2.75 | | 3.58 | | | | | 0.63 | | | 0.56 | | | | 0.72 | |
| 11 | Oregon | | | | | | | 2,790 | | | | 430 | | 226 | | | 1,555 | | | 579 | | | 0.43 | | | | 0.40 | | | 0.46 | | 0.87 | | | | 0.86 | | | 0.89 | | | | 0.08 | | | 0.98 | | | 3.36 | | | | 2.92 | | 3.87 | | | | | 0.66 | | | 0.58 | | | | 0.75 | |
| 12 | PHI | | | | | | | 2,790 | | | | 227 | | 121 | | | 1,660 | | | 782 | | | 0.22 | | | | 0.20 | | | 0.25 | | 0.93 | | | | 0.92 | | | 0.94 | | | | 0.08 | | | 0.98 | | | 3.31 | | | | 2.69 | | 4.07 | | | | | 0.83 | | | 0.70 | | | | 0.99 | |
| 13 | RTST | | | | | | | 2,790 | | | | 369 | | 202 | | | 1,579 | | | 640 | | | 0.37 | | | | 0.34 | | | 0.40 | | 0.89 | | | | 0.87 | | | 0.90 | | | | 0.08 | | | 0.98 | | | 3.22 | | | | 2.77 | | 3.76 | | | | | 0.72 | | | 0.62 | | | | 0.82 | |
| 14 | SWAS | | | | | | | 2,790 | | | | 374 | | 158 | | | 1,623 | | | 635 | | | 0.37 | | | | 0.34 | | | 0.40 | | 0.91 | | | | 0.90 | | | 0.92 | | | | 0.10 | | | 0.98 | | | 4.18 | | | | 3.53 | | 4.95 | | | | | 0.69 | | | 0.59 | | | | 0.81 | |
| 15 | Trauma Score | | | | | | | 2,790 | | | | 148 | | 50 | | | 1,731 | | | 861 | | | 0.15 | | | | 0.12 | | | 0.17 | | 0.97 | | | | 0.96 | | | 0.98 | | | | 0.11 | | | 0.98 | | | 5.22 | | | | 3.83 | | 7.13 | | | | | 0.88 | | | 0.67 | | | | 1.16 | |
| 16 | Trauma Scorecard | | | | | | | 2,790 | | | | 397 | | 211 | | | 1,570 | | | 612 | | | 0.39 | | | | 0.36 | | | 0.42 | | 0.88 | | | | 0.87 | | | 0.90 | | | | 0.08 | | | 0.98 | | | 3.32 | | | | 2.86 | | 3.85 | | | | | 0.69 | | | 0.60 | | | | 0.79 | |
| 17 | TTR | | | | | | | 2,790 | | | | 260 | | 132 | | | 1,649 | | | 749 | | | 0.26 | | | | 0.23 | | | 0.28 | | 0.93 | | | | 0.91 | | | 0.94 | | | | 0.08 | | | 0.98 | | | 3.48 | | | | 2.86 | | 4.22 | | | | | 0.80 | | | 0.68 | | | | 0.95 | |
| 18 | US Field Triage | | | | | | | 2,790 | | | | 480 | | 286 | | | 1,495 | | | 529 | | | 0.48 | | | | 0.44 | | | 0.51 | | 0.84 | | | | 0.82 | | | 0.86 | | | | 0.07 | | | 0.98 | | | 2.96 | | | | 2.62 | | 3.35 | | | | | 0.62 | | | 0.55 | | | | 0.71 | |
| 19 | Victoria | | | | | | | 2,790 | | | | 668 | | 552 | | | 1,229 | | | 341 | | | 0.66 | | | | 0.63 | | | 0.69 | | 0.69 | | | | 0.67 | | | 0.71 | | | | 0.05 | | | 0.99 | | | 2.14 | | | | 1.97 | | 2.32 | | | | | 0.49 | | | 0.44 | | | | 0.55 | |
| 20 | Vittel | | | | | | | 2,790 | | | | 533 | | 331 | | | 1,450 | | | 476 | | | 0.53 | | | | 0.50 | | | 0.56 | | 0.81 | | | | 0.80 | | | 0.83 | | | | 0.07 | | | 0.99 | | | 2.84 | | | | 2.54 | | 3.18 | | | | | 0.58 | | | 0.52 | | | | 0.65 | |
| 21 | WMAS | | | | | | | 2,790 | | | | 504 | | 285 | | | 1,496 | | | 505 | | | 0.50 | | | | 0.47 | | | 0.53 | | 0.84 | | | | 0.82 | | | 0.86 | | | | 0.08 | | | 0.98 | | | 3.12 | | | | 2.76 | | 3.53 | | | | | 0.60 | | | 0.53 | | | | 0.67 | |
| 22 | YAS | | | | | | | 2,790 | | | | 557 | | 318 | | | 1,463 | | | 452 | | | 0.55 | | | | 0.52 | | | 0.58 | | 0.82 | | | | 0.80 | | | 0.84 | | | | 0.08 | | | 0.99 | | | 3.09 | | | | 2.76 | | 3.47 | | | | | 0.55 | | | 0.48 | | | | 0.62 | |
| **MISSING AT RANDOM MISSING DATA SCENARIO (MULTIPLE IMPUTATION)** | | | | | | | | | | | | | | | | | | | | | | | | | | | | | | | | | | | | | | | | | | | | | | | | | | | | | | | | | | | | | | | | | | | | |
| Tool number | | | | Tool name | | | | | | | | | | | | | | | | | | | | Sensitivity | | | | | | | | | Sens LCL | | | | | | | Sens UCL | | | | | | | | | | Specificity | | | | | | | Spec LCL | | | | | | | Spec UCL | | | | |
| 1 | | | | CRAMS | | | | | | | | | | | | | | | | | | | | 0.35 | | | | | | | | | 0.32 | | | | | | | 0.38 | | | | | | | | | | 0.93 | | | | | | | 0.92 | | | | | | | 0.94 | | | | |
| 2 | | | | Dutch | | | | | | | | | | | | | | | | | | | | 0.29 | | | | | | | | | 0.26 | | | | | | | 0.32 | | | | | | | | | | 0.93 | | | | | | | 0.92 | | | | | | | 0.94 | | | | |
| 3 | | | | Florida | | | | | | | | | | | | | | | | | | | | 0.43 | | | | | | | | | 0.40 | | | | | | | 0.46 | | | | | | | | | | 0.86 | | | | | | | 0.84 | | | | | | | 0.87 | | | | |
| 4 | | | | LAS (current) | | | | | | | | | | | | | | | | | | | | 0.46 | | | | | | | | | 0.43 | | | | | | | 0.49 | | | | | | | | | | 0.89 | | | | | | | 0.88 | | | | | | | 0.91 | | | | |
| 5 | | | | LAS (old) | | | | | | | | | | | | | | | | | | | | 0.57 | | | | | | | | | 0.54 | | | | | | | 0.60 | | | | | | | | | | 0.86 | | | | | | | 0.84 | | | | | | | 0.87 | | | | |
| 6 | | | | MATTS balanced | | | | | | | | | | | | | | | | | | | | 0.59 | | | | | | | | | 0.56 | | | | | | | 0.62 | | | | | | | | | | 0.86 | | | | | | | 0.85 | | | | | | | 0.88 | | | | |
| 7 | | | | MATTS sensitive | | | | | | | | | | | | | | | | | | | | 0.73 | | | | | | | | | 0.70 | | | | | | | 0.76 | | | | | | | | | | 0.75 | | | | | | | 0.73 | | | | | | | 0.77 | | | | |
| 8 | | | | MATTS specific | | | | | | | | | | | | | | | | | | | | 0.39 | | | | | | | | | 0.36 | | | | | | | 0.42 | | | | | | | | | | 0.95 | | | | | | | 0.94 | | | | | | | 0.96 | | | | |
| 9 | | | | MGAP | | | | | | | | | | | | | | | | | | | | 0.32 | | | | | | | | | 0.29 | | | | | | | 0.35 | | | | | | | | | | 0.85 | | | | | | | 0.83 | | | | | | | 0.86 | | | | |
| 10 | | | | North Carolina | | | | | | | | | | | | | | | | | | | | 0.43 | | | | | | | | | 0.40 | | | | | | | 0.46 | | | | | | | | | | 0.89 | | | | | | | 0.88 | | | | | | | 0.91 | | | | |
| 11 | | | | Oregon | | | | | | | | | | | | | | | | | | | | 0.40 | | | | | | | | | 0.37 | | | | | | | 0.43 | | | | | | | | | | 0.91 | | | | | | | 0.90 | | | | | | | 0.92 | | | | |
| 12 | | | | PHI | | | | | | | | | | | | | | | | | | | | 0.20 | | | | | | | | | 0.18 | | | | | | | 0.23 | | | | | | | | | | 0.96 | | | | | | | 0.95 | | | | | | | 0.97 | | | | |
| 13 | | | | RTST | | | | | | | | | | | | | | | | | | | | 0.33 | | | | | | | | | 0.30 | | | | | | | 0.36 | | | | | | | | | | 0.93 | | | | | | | 0.91 | | | | | | | 0.94 | | | | |
| 14 | | | | SWAS | | | | | | | | | | | | | | | | | | | | 0.34 | | | | | | | | | 0.31 | | | | | | | 0.37 | | | | | | | | | | 0.95 | | | | | | | 0.94 | | | | | | | 0.96 | | | | |
| 15 | | | | Trauma Score | | | | | | | | | | | | | | | | | | | | 0.11 | | | | | | | | | 0.09 | | | | | | | 0.13 | | | | | | | | | | 0.99 | | | | | | | 0.98 | | | | | | | 0.99 | | | | |
| 16 | | | | Trauma Scorecard | | | | | | | | | | | | | | | | | | | | 0.36 | | | | | | | | | 0.33 | | | | | | | 0.39 | | | | | | | | | | 0.92 | | | | | | | 0.91 | | | | | | | 0.93 | | | | |
| 17 | | | | TTR | | | | | | | | | | | | | | | | | | | | 0.22 | | | | | | | | | 0.20 | | | | | | | 0.25 | | | | | | | | | | 0.96 | | | | | | | 0.95 | | | | | | | 0.97 | | | | |
| 18 | | | | US Field Triage | | | | | | | | | | | | | | | | | | | | 0.45 | | | | | | | | | 0.42 | | | | | | | 0.48 | | | | | | | | | | 0.88 | | | | | | | 0.86 | | | | | | | 0.89 | | | | |
| 19 | | | | Victoria | | | | | | | | | | | | | | | | | | | | 0.62 | | | | | | | | | 0.59 | | | | | | | 0.65 | | | | | | | | | | 0.75 | | | | | | | 0.72 | | | | | | | 0.77 | | | | |
| 20 | | | | Vittel | | | | | | | | | | | | | | | | | | | | 0.48 | | | | | | | | | 0.45 | | | | | | | 0.51 | | | | | | | | | | 0.87 | | | | | | | 0.85 | | | | | | | 0.89 | | | | |
| 21 | | | | WMAS | | | | | | | | | | | | | | | | | | | | 0.48 | | | | | | | | | 0.45 | | | | | | | 0.51 | | | | | | | | | | 0.88 | | | | | | | 0.86 | | | | | | | 0.89 | | | | |
| 22 | | | | YAS | | | | | | | | | | | | | | | | | | | | 0.53 | | | | | | | | | 0.49 | | | | | | | 0.56 | | | | | | | | | | 0.86 | | | | | | | 0.84 | | | | | | | 0.87 | | | | |

CRAMS: Circulation, Respiration, Abdomen, Motor, and Speech Scale; Dutch: Dutch Field Triage Protocol; Florida: State of Florida Trauma Criteria; LAS (current): London Ambulance Service (current) Major Trauma Triage Tool; LAS (old): London Ambulance Service (old) Major Trauma Triage Tool; MATTS balanced: Newly developed MATTS triage tool – balancing sensitivity/specificity; MATTS sensitive: Newly developed MATTS triage tool – prioritising sensitivity; MATTS specific: Newly developed MATTS triage tool – prioritising specificity; MGAP: Mechanism, Glasgow Coma Scale, Age, and Arterial Pressure Score; North Carolina: North Carolina Trauma and Burn EMS Triage and Destination Plan; Oregon: Oregon Guidelines for Field Triage of Injured Patients; PHI: The Prehospital Index; RTST: Triage Revised Trauma Score; SWAS: South West Ambulance Service Major Trauma Triage Tool; Trauma Score: Trauma Score; Trauma Scorecard: Trauma Scorecard; TTR: Trauma Triage Rule; US Field Triage: National Guidelines for the Field Triage of Injured Patients (2011); Victoria: Pre-hospital Major Trauma Triage - Trauma Victoria; Vittel: Vittel criteria for severe trauma triage; WMAS: West Midlands Ambulance Service Major Trauma Triage Tool: YAS Yorkshire Ambulance Service Major Trauma Triage Tool
