# Supplementary material for: Major Trauma Triage Study (MATTS): Diagnostic accuracy of major trauma triage tools in English regional trauma networks – A case-cohort study
Source: PLoS One. 2026 Mar 27;21(3):e0344996. doi: 10.1371/journal.pone.0344996 (PMC13029787; doi:10.1371/journal.pone.0344996)
Supplement: S5 Table — (DOCX) [file pone.0344996.s005.docx]

**SUPPLEMENTARY MATERIALS S5**

**Diagnostic accuracy metrics for selected triage tools evaluated against the primary MATTS reference standard in different subgroups**

| **Patients aged between 16 and 64 years** | | | | | | | | | | | | | | | | | | | | |
| --- | --- | --- | --- | --- | --- | --- | --- | --- | --- | --- | --- | --- | --- | --- | --- | --- | --- | --- | --- | --- |
| Tool number | Tool name | n | TP | FP | TN | FN | Sensitivity | Sens LCL | Sens UCL | Specificity | Spec LCL | Spec UCL | PPV | NPV | Positive LR | Positive LR LCL | PositiveLRucl | NegativeLR | Negative LR LCL | Negative LR UCL |
| 1 | CRAMS | 1,135 | 198 | 44 | 600 | 293 | 0.40 | 0.36 | 0.45 | 0.93 | 0.91 | 0.95 | 0.19 | 0.98 | 5.90 | 4.35 | 8.01 | 0.64 | 0.48 | 0.86 |
| 2 | Dutch | 1,135 | 179 | 76 | 568 | 312 | 0.36 | 0.32 | 0.41 | 0.88 | 0.86 | 0.91 | 0.11 | 0.97 | 3.09 | 2.43 | 3.93 | 0.72 | 0.58 | 0.90 |
| 3 | Florida | 1,135 | 251 | 119 | 525 | 240 | 0.51 | 0.47 | 0.56 | 0.82 | 0.79 | 0.85 | 0.10 | 0.98 | 2.77 | 2.30 | 3.32 | 0.60 | 0.50 | 0.72 |
| 4 | LAS (current) | 1,135 | 278 | 90 | 554 | 213 | 0.57 | 0.52 | 0.61 | 0.86 | 0.83 | 0.89 | 0.13 | 0.98 | 4.05 | 3.29 | 4.98 | 0.50 | 0.41 | 0.63 |
| 5 | LAS (old) | 1,135 | 326 | 114 | 530 | 165 | 0.66 | 0.62 | 0.71 | 0.82 | 0.79 | 0.85 | 0.12 | 0.99 | 3.75 | 3.14 | 4.48 | 0.41 | 0.33 | 0.50 |
| 6 | MATTS balanced | 1,135 | 319 | 101 | 543 | 172 | 0.65 | 0.61 | 0.69 | 0.84 | 0.82 | 0.87 | 0.13 | 0.99 | 4.14 | 3.42 | 5.01 | 0.42 | 0.33 | 0.52 |
| 7 | MATTS sensitive | 1,135 | 400 | 208 | 436 | 91 | 0.81 | 0.78 | 0.85 | 0.68 | 0.64 | 0.71 | 0.08 | 0.99 | 2.52 | 2.24 | 2.84 | 0.27 | 0.22 | 0.34 |
| 8 | MATTS specific | 1,135 | 234 | 48 | 596 | 257 | 0.48 | 0.43 | 0.52 | 0.93 | 0.91 | 0.95 | 0.19 | 0.98 | 6.39 | 4.80 | 8.52 | 0.57 | 0.43 | 0.75 |
| 9 | MGAP | 1,135 | 125 | 31 | 613 | 366 | 0.25 | 0.22 | 0.29 | 0.95 | 0.94 | 0.97 | 0.18 | 0.97 | 5.29 | 3.63 | 7.70 | 0.78 | 0.55 | 1.11 |
| 10 | North Carolina | 1,135 | 267 | 88 | 556 | 224 | 0.54 | 0.50 | 0.59 | 0.86 | 0.84 | 0.89 | 0.13 | 0.98 | 3.98 | 3.22 | 4.91 | 0.53 | 0.43 | 0.66 |
| 11 | Oregon | 1,135 | 241 | 68 | 576 | 250 | 0.49 | 0.45 | 0.54 | 0.89 | 0.87 | 0.92 | 0.16 | 0.98 | 4.65 | 3.65 | 5.92 | 0.57 | 0.45 | 0.72 |
| 12 | PHI | 1,135 | 121 | 28 | 616 | 370 | 0.25 | 0.21 | 0.28 | 0.96 | 0.94 | 0.97 | 0.17 | 0.97 | 5.67 | 3.82 | 8.40 | 0.79 | 0.55 | 1.14 |
| 13 | RTST | 1,135 | 193 | 47 | 597 | 298 | 0.39 | 0.35 | 0.44 | 0.93 | 0.91 | 0.95 | 0.19 | 0.98 | 5.39 | 4.00 | 7.24 | 0.65 | 0.49 | 0.87 |
| 14 | SWAS | 1,135 | 203 | 39 | 605 | 288 | 0.41 | 0.37 | 0.46 | 0.94 | 0.92 | 0.96 | 0.20 | 0.98 | 6.83 | 4.95 | 9.42 | 0.62 | 0.46 | 0.85 |
| 15 | Trauma Score | 1,135 | 65 | 7 | 637 | 426 | 0.13 | 0.10 | 0.16 | 0.99 | 0.98 | 1.00 | 0.32 | 0.97 | 12.18 | 5.63 | 26.32 | 0.88 | 0.42 | 1.83 |
| 16 | Trauma Scorecard | 1,135 | 225 | 66 | 578 | 266 | 0.46 | 0.41 | 0.50 | 0.90 | 0.87 | 0.92 | 0.15 | 0.98 | 4.47 | 3.49 | 5.73 | 0.60 | 0.47 | 0.77 |
| 17 | TTR | 1,135 | 145 | 39 | 605 | 346 | 0.30 | 0.25 | 0.34 | 0.94 | 0.92 | 0.96 | 0.17 | 0.97 | 4.88 | 3.49 | 6.81 | 0.75 | 0.55 | 1.02 |
| 18 | US Field Triage | 1,135 | 269 | 98 | 546 | 222 | 0.55 | 0.50 | 0.59 | 0.85 | 0.82 | 0.88 | 0.12 | 0.98 | 3.60 | 2.95 | 4.39 | 0.53 | 0.43 | 0.66 |
| 19 | Victoria | 1,135 | 343 | 183 | 461 | 148 | 0.70 | 0.66 | 0.74 | 0.72 | 0.68 | 0.75 | 0.08 | 0.98 | 2.46 | 2.15 | 2.82 | 0.42 | 0.35 | 0.51 |
| 20 | Vittel | 1,135 | 281 | 88 | 556 | 210 | 0.57 | 0.53 | 0.62 | 0.86 | 0.84 | 0.89 | 0.14 | 0.98 | 4.19 | 3.40 | 5.16 | 0.50 | 0.40 | 0.62 |
| 21 | WMAS | 1,135 | 279 | 96 | 548 | 212 | 0.57 | 0.52 | 0.61 | 0.85 | 0.82 | 0.88 | 0.13 | 0.98 | 3.81 | 3.12 | 4.66 | 0.51 | 0.41 | 0.63 |
| 22 | YAS | 1,135 | 291 | 114 | 530 | 200 | 0.59 | 0.55 | 0.64 | 0.82 | 0.79 | 0.85 | 0.11 | 0.98 | 3.35 | 2.79 | 4.02 | 0.49 | 0.41 | 0.60 |

| **Patients aged over 65 years.** | | | | | | | | | | | | | | | | | | | | | | | | | | | | | | | | | | | | | | | | | | | | | | | | | | | | |
| --- | --- | --- | --- | --- | --- | --- | --- | --- | --- | --- | --- | --- | --- | --- | --- | --- | --- | --- | --- | --- | --- | --- | --- | --- | --- | --- | --- | --- | --- | --- | --- | --- | --- | --- | --- | --- | --- | --- | --- | --- | --- | --- | --- | --- | --- | --- | --- | --- | --- | --- | --- | --- |
| **Tool Number** | | | **Tool Name** | | | | | **n** | | | **Sensitivity** | | | | **LCL** | | **UCL** | | | **Specificity** | | | | **LCL** | | **UCL** | | | **Accuracy** | | | **TP** | | | **FP** | | | **TN** | | | **FN** | | | **PPV** | | | **NPV** | | **Positive LR** | | **Negative LR** | |
| 1 | | | CRAMS | | | | | 1472 | | | 0.26 | | | | 0.22 | | 0.30 | | | 0.94 | | | | 0.92 | | 0.95 | | | 0.74 | | | 112.00 | | | 63.00 | | | 972.00 | | | 325.00 | | | 0.07 | | | 0.99 | | 4.21 | | 0.79 | |
| 2 | | | Dutch | | | | | 1472 | | | 0.16 | | | | 0.13 | | 0.20 | | | 0.96 | | | | 0.95 | | 0.98 | | | 0.73 | | | 72.00 | | | 37.00 | | | 998.00 | | | 365.00 | | | 0.07 | | | 0.98 | | 4.61 | | 0.87 | |
| 3 | | | Florida | | | | | 1472 | | | 0.29 | | | | 0.25 | | 0.34 | | | 0.88 | | | | 0.86 | | 0.90 | | | 0.71 | | | 128.00 | | | 120.00 | | | 915.00 | | | 309.00 | | | 0.04 | | | 0.99 | | 2.53 | | 0.80 | |
| 4 | | | LAS (current) | | | | | 1472 | | | 0.31 | | | | 0.27 | | 0.35 | | | 0.92 | | | | 0.90 | | 0.93 | | | 0.74 | | | 136.00 | | | 86.00 | | | 949.00 | | | 301.00 | | | 0.07 | | | 0.99 | | 3.75 | | 0.75 | |
| 5 | | | LAS (old) | | | | | 1472 | | | 0.44 | | | | 0.39 | | 0.49 | | | 0.88 | | | | 0.86 | | 0.90 | | | 0.75 | | | 192.00 | | | 124.00 | | | 911.00 | | | 245.00 | | | 0.07 | | | 0.99 | | 3.67 | | 0.64 | |
| 6 | | | MATTS balanced | | | | | 1472 | | | 0.50 | | | | 0.45 | | 0.55 | | | 0.89 | | | | 0.87 | | 0.91 | | | 0.77 | | | 219.00 | | | 118.00 | | | 917.00 | | | 218.00 | | | 0.08 | | | 0.99 | | 4.40 | | 0.56 | |
| 7 | | | MATTS sensitive | | | | | 1472 | | | 0.62 | | | | 0.58 | | 0.67 | | | 0.81 | | | | 0.78 | | 0.83 | | | 0.75 | | | 272.00 | | | 199.00 | | | 836.00 | | | 165.00 | | | 0.06 | | | 0.99 | | 3.24 | | 0.47 | |
| 8 | | | MATTS specific | | | | | 1472 | | | 0.26 | | | | 0.22 | | 0.30 | | | 0.97 | | | | 0.96 | | 0.98 | | | 0.76 | | | 112.00 | | | 33.00 | | | 1002.00 | | | 325.00 | | | 0.14 | | | 0.99 | | 8.04 | | 0.77 | |
| 9 | | | MGAP | | | | | 1472 | | | 0.38 | | | | 0.33 | | 0.42 | | | 0.79 | | | | 0.76 | | 0.81 | | | 0.66 | | | 164.00 | | | 222.00 | | | 813.00 | | | 273.00 | | | 0.03 | | | 0.99 | | 1.75 | | 0.80 | |
| 10 | | | North Carolina | | | | | 1472 | | | 0.27 | | | | 0.23 | | 0.31 | | | 0.91 | | | | 0.89 | | 0.93 | | | 0.72 | | | 118.00 | | | 93.00 | | | 942.00 | | | 319.00 | | | 0.05 | | | 0.99 | | 3.01 | | 0.80 | |
| 11 | | | Oregon | | | | | 1472 | | | 0.25 | | | | 0.21 | | 0.29 | | | 0.92 | | | | 0.91 | | 0.94 | | | 0.72 | | | 109.00 | | | 78.00 | | | 957.00 | | | 328.00 | | | 0.06 | | | 0.99 | | 3.31 | | 0.81 | |
| 12 | | | PHI | | | | | 1472 | | | 0.11 | | | | 0.08 | | 0.14 | | | 0.96 | | | | 0.95 | | 0.97 | | | 0.71 | | | 47.00 | | | 38.00 | | | 997.00 | | | 390.00 | | | 0.05 | | | 0.98 | | 2.93 | | 0.93 | |
| 13 | | | RTST | | | | | 1472 | | | 0.22 | | | | 0.18 | | 0.26 | | | 0.93 | | | | 0.91 | | 0.94 | | | 0.72 | | | 96.00 | | | 74.00 | | | 961.00 | | | 341.00 | | | 0.05 | | | 0.98 | | 3.07 | | 0.84 | |
| 14 | | | SWAS | | | | | 1472 | | | 0.22 | | | | 0.18 | | 0.26 | | | 0.96 | | | | 0.95 | | 0.97 | | | 0.74 | | | 97.00 | | | 41.00 | | | 994.00 | | | 340.00 | | | 0.10 | | | 0.99 | | 5.60 | | 0.81 | |
| 15 | | | Trauma Score | | | | | 1472 | | | 0.06 | | | | 0.04 | | 0.08 | | | 0.99 | | | | 0.99 | | 1.00 | | | 0.72 | | | 27.00 | | | 7.00 | | | 1028.00 | | | 410.00 | | | 0.18 | | | 0.98 | | 9.14 | | 0.95 | |
| 16 | | | Trauma Scorecard | | | | | 1472 | | | 0.21 | | | | 0.17 | | 0.25 | | | 0.94 | | | | 0.92 | | 0.95 | | | 0.72 | | | 93.00 | | | 65.00 | | | 970.00 | | | 344.00 | | | 0.06 | | | 0.98 | | 3.39 | | 0.84 | |
| 17 | | | TTR | | | | | 1472 | | | 0.11 | | | | 0.08 | | 0.14 | | | 0.97 | | | | 0.96 | | 0.98 | | | 0.72 | | | 48.00 | | | 27.00 | | | 1008.00 | | | 389.00 | | | 0.07 | | | 0.98 | | 4.21 | | 0.91 | |
| 18 | | | US Field Triage | | | | | 1472 | | | 0.30 | | | | 0.26 | | 0.35 | | | 0.90 | | | | 0.88 | | 0.91 | | | 0.72 | | | 132.00 | | | 108.00 | | | 927.00 | | | 305.00 | | | 0.05 | | | 0.99 | | 2.90 | | 0.78 | |
| 19 | | | Victoria | | | | | 1472 | | | 0.49 | | | | 0.45 | | 0.54 | | | 0.77 | | | | 0.74 | | 0.79 | | | 0.69 | | | 216.00 | | | 239.00 | | | 796.00 | | | 221.00 | | | 0.04 | | | 0.99 | | 2.14 | | 0.66 | |
| 20 | | | Vittel | | | | | 1472 | | | 0.31 | | | | 0.27 | | 0.35 | | | 0.88 | | | | 0.86 | | 0.90 | | | 0.71 | | | 136.00 | | | 123.00 | | | 912.00 | | | 301.00 | | | 0.04 | | | 0.99 | | 2.62 | | 0.78 | |
| 21 | | | WMAS | | | | | 1472 | | | 0.33 | | | | 0.29 | | 0.38 | | | 0.89 | | | | 0.88 | | 0.91 | | | 0.73 | | | 146.00 | | | 109.00 | | | 926.00 | | | 291.00 | | | 0.06 | | | 0.99 | | 3.17 | | 0.74 | |
| 22 | | | YAS | | | | | 1472 | | | 0.42 | | | | 0.38 | | 0.47 | | | 0.88 | | | | 0.86 | | 0.90 | | | 0.74 | | | 185.00 | | | 124.00 | | | 911.00 | | | 252.00 | | | 0.07 | | | 0.99 | | 3.53 | | 0.66 | |
| **Blunt trauma** | | | | | | | | | | | | | | | | | | | | | | | | | | | | | | | | | | | | | | | | | | | | | | | | | | | | |
| **Tool Number** | | **Tool Name** | | | | **n** | | | **Sensitivity** | | | | **LCL** | | | **UCL** | | | **Specificity** | | | **LCL** | | | **UCL** | | | **Accuracy** | | | **TP** | | | **FP** | | | **TN** | | | **FN** | | **PPV** | | | **NPV** | | | **Positive LR** | | | **Negative LR** | |
| 1 | | CRAMS | | | | 2514 | | | 0.31 | | | | 0.28 | | | 0.34 | | | 0.94 | | | 0.93 | | | 0.95 | | | 0.72 | | | 267 | | | 93 | | | 1548 | | | 606 | | 0.12 | | | 0.98 | | | 5.40 | | | 0.74 | |
| 2 | | Dutch | | | | 2514 | | | 0.23 | | | | 0.21 | | | 0.26 | | | 0.94 | | | 0.93 | | | 0.95 | | | 0.70 | | | 205 | | | 95 | | | 1546 | | | 668 | | 0.09 | | | 0.98 | | | 4.06 | | | 0.81 | |
| 3 | | Florida | | | | 2514 | | | 0.38 | | | | 0.34 | | | 0.41 | | | 0.87 | | | 0.85 | | | 0.89 | | | 0.70 | | | 328 | | | 214 | | | 1427 | | | 545 | | 0.07 | | | 0.98 | | | 2.88 | | | 0.72 | |
| 4 | | LAS (current) | | | | 2514 | | | 0.42 | | | | 0.39 | | | 0.45 | | | 0.91 | | | 0.89 | | | 0.92 | | | 0.74 | | | 367 | | | 151 | | | 1490 | | | 506 | | 0.10 | | | 0.98 | | | 4.57 | | | 0.64 | |
| 5 | | LAS (old) | | | | 2514 | | | 0.54 | | | | 0.50 | | | 0.57 | | | 0.87 | | | 0.85 | | | 0.88 | | | 0.75 | | | 469 | | | 219 | | | 1422 | | | 404 | | 0.09 | | | 0.99 | | | 4.03 | | | 0.53 | |
| 6 | | MATTS balanced | | | | 2514 | | | 0.56 | | | | 0.52 | | | 0.59 | | | 0.88 | | | 0.86 | | | 0.90 | | | 0.77 | | | 487 | | | 196 | | | 1445 | | | 386 | | 0.10 | | | 0.99 | | | 4.67 | | | 0.50 | |
| 7 | | MATTS sensitive | | | | 2514 | | | 0.71 | | | | 0.68 | | | 0.74 | | | 0.77 | | | 0.75 | | | 0.79 | | | 0.75 | | | 619 | | | 381 | | | 1260 | | | 254 | | 0.07 | | | 0.99 | | | 3.05 | | | 0.38 | |
| 8 | | MATTS specific | | | | 2514 | | | 0.34 | | | | 0.31 | | | 0.37 | | | 0.96 | | | 0.95 | | | 0.97 | | | 0.75 | | | 298 | | | 63 | | | 1578 | | | 575 | | 0.18 | | | 0.98 | | | 8.89 | | | 0.69 | |
| 9 | | MGAP | | | | 2514 | | | 0.31 | | | | 0.28 | | | 0.34 | | | 0.85 | | | 0.83 | | | 0.87 | | | 0.66 | | | 272 | | | 245 | | | 1396 | | | 601 | | 0.05 | | | 0.98 | | | 2.09 | | | 0.81 | |
| 10 | | North Carolina | | | | 2514 | | | 0.38 | | | | 0.35 | | | 0.41 | | | 0.91 | | | 0.89 | | | 0.92 | | | 0.72 | | | 332 | | | 153 | | | 1488 | | | 541 | | 0.09 | | | 0.98 | | | 4.08 | | | 0.68 | |
| 11 | | Oregon | | | | 2514 | | | 0.34 | | | | 0.31 | | | 0.38 | | | 0.93 | | | 0.91 | | | 0.94 | | | 0.72 | | | 301 | | | 120 | | | 1521 | | | 572 | | 0.11 | | | 0.98 | | | 4.72 | | | 0.71 | |
| 12 | | PHI | | | | 2514 | | | 0.14 | | | | 0.12 | | | 0.17 | | | 0.97 | | | 0.96 | | | 0.98 | | | 0.68 | | | 124 | | | 53 | | | 1588 | | | 749 | | 0.09 | | | 0.98 | | | 4.40 | | | 0.89 | |
| 13 | | RTST | | | | 2514 | | | 0.30 | | | | 0.27 | | | 0.33 | | | 0.93 | | | 0.92 | | | 0.94 | | | 0.71 | | | 263 | | | 116 | | | 1525 | | | 610 | | 0.10 | | | 0.98 | | | 4.26 | | | 0.75 | |
| 14 | | SWAS | | | | 2514 | | | 0.32 | | | | 0.29 | | | 0.35 | | | 0.95 | | | 0.94 | | | 0.96 | | | 0.73 | | | 279 | | | 76 | | | 1565 | | | 594 | | 0.15 | | | 0.98 | | | 6.90 | | | 0.71 | |
| 15 | | Trauma Score | | | | 2514 | | | 0.10 | | | | 0.08 | | | 0.11 | | | 0.99 | | | 0.99 | | | 1.00 | | | 0.68 | | | 83 | | | 14 | | | 1627 | | | 790 | | 0.24 | | | 0.98 | | | 11.14 | | | 0.91 | |
| 16 | | Trauma Scorecard | | | | 2514 | | | 0.31 | | | | 0.28 | | | 0.34 | | | 0.93 | | | 0.92 | | | 0.95 | | | 0.72 | | | 269 | | | 109 | | | 1532 | | | 604 | | 0.10 | | | 0.98 | | | 4.64 | | | 0.74 | |
| 17 | | TTR | | | | 2514 | | | 0.17 | | | | 0.14 | | | 0.19 | | | 0.97 | | | 0.97 | | | 0.98 | | | 0.69 | | | 145 | | | 44 | | | 1597 | | | 728 | | 0.13 | | | 0.98 | | | 6.20 | | | 0.86 | |
| 18 | | US Field Triage | | | | 2514 | | | 0.40 | | | | 0.37 | | | 0.43 | | | 0.89 | | | 0.88 | | | 0.91 | | | 0.72 | | | 350 | | | 178 | | | 1463 | | | 523 | | 0.08 | | | 0.98 | | | 3.70 | | | 0.67 | |
| 19 | | Victoria | | | | 2514 | | | 0.58 | | | | 0.55 | | | 0.61 | | | 0.77 | | | 0.74 | | | 0.79 | | | 0.70 | | | 508 | | | 385 | | | 1256 | | | 365 | | 0.05 | | | 0.99 | | | 2.48 | | | 0.55 | |
| 20 | | Vittel | | | | 2514 | | | 0.42 | | | | 0.39 | | | 0.45 | | | 0.89 | | | 0.88 | | | 0.91 | | | 0.73 | | | 366 | | | 178 | | | 1463 | | | 507 | | 0.08 | | | 0.98 | | | 3.87 | | | 0.65 | |
| 21 | | WMAS | | | | 2514 | | | 0.43 | | | | 0.40 | | | 0.46 | | | 0.89 | | | 0.88 | | | 0.91 | | | 0.73 | | | 374 | | | 180 | | | 1461 | | | 499 | | 0.09 | | | 0.98 | | | 3.91 | | | 0.64 | |
| 22 | | YAS | | | | 2514 | | | 0.49 | | | | 0.46 | | | 0.52 | | | 0.87 | | | 0.85 | | | 0.88 | | | 0.74 | | | 427 | | | 219 | | | 1422 | | | 446 | | 0.08 | | | 0.99 | | | 3.67 | | | 0.59 | |
| **Penetrating trauma** | | | | | | | | | | | | | | | | | | | | | | | | | | | | | | | | | | | | | | | | | | | | | | | | | | | | |
| **Tool Number** | | **Tool Name** | | | | **n** | | | **Sensitivity** | | | | **LCL** | | | **UCL** | | | **Specificity** | | | **LCL** | | | **UCL** | | | **Accuracy** | | | **TP** | | | **FP** | | | **TN** | | | **FN** | | **PPV** | | | **NPV** | | | **Positive LR** | | | **Negative LR** | |
| 1 | | CRAMS | | | | 93 | | | 0.78 | | | | 0.67 | | | 0.89 | | | 0.63 | | | 0.48 | | | 0.78 | | | 0.72 | | | 43 | | | 14 | | | 24 | | | 12 | | 0.15 | | | 0.97 | | | 2.09 | | | 0.36 | |
| 2 | | Dutch | | | | 93 | | | 0.84 | | | | 0.74 | | | 0.93 | | | 0.53 | | | 0.37 | | | 0.69 | | | 0.71 | | | 46 | | | 18 | | | 20 | | | 9 | | 0.12 | | | 0.97 | | | 1.75 | | | 0.32 | |
| 3 | | Florida | | | | 93 | | | 0.93 | | | | 0.86 | | | 1.00 | | | 0.34 | | | 0.19 | | | 0.49 | | | 0.69 | | | 51 | | | 25 | | | 13 | | | 4 | | 0.10 | | | 0.98 | | | 1.41 | | | 0.23 | |
| 4 | | LAS (current) | | | | 93 | | | 0.85 | | | | 0.76 | | | 0.95 | | | 0.34 | | | 0.19 | | | 0.49 | | | 0.65 | | | 47 | | | 25 | | | 13 | | | 8 | | 0.10 | | | 0.97 | | | 1.30 | | | 0.44 | |
| 5 | | LAS (old) | | | | 93 | | | 0.89 | | | | 0.81 | | | 0.97 | | | 0.50 | | | 0.34 | | | 0.66 | | | 0.73 | | | 49 | | | 19 | | | 19 | | | 6 | | 0.13 | | | 0.98 | | | 1.77 | | | 0.23 | |
| 6 | | MATTS balanced | | | | 93 | | | 0.93 | | | | 0.86 | | | 1.00 | | | 0.39 | | | 0.24 | | | 0.55 | | | 0.71 | | | 51 | | | 23 | | | 15 | | | 4 | | 0.11 | | | 0.98 | | | 1.53 | | | 0.20 | |
| 7 | | MATTS sensitive | | | | 93 | | | 0.96 | | | | 0.91 | | | 1.01 | | | 0.32 | | | 0.17 | | | 0.46 | | | 0.70 | | | 53 | | | 26 | | | 12 | | | 2 | | 0.11 | | | 0.99 | | | 1.41 | | | 0.14 | |
| 8 | | MATTS specific | | | | 93 | | | 0.87 | | | | 0.78 | | | 0.96 | | | 0.53 | | | 0.37 | | | 0.69 | | | 0.73 | | | 48 | | | 18 | | | 20 | | | 7 | | 0.14 | | | 0.98 | | | 1.83 | | | 0.26 | |
| 9 | | MGAP | | | | 93 | | | 0.31 | | | | 0.19 | | | 0.43 | | | 0.79 | | | 0.66 | | | 0.92 | | | 0.51 | | | 17 | | | 8 | | | 30 | | | 38 | | 0.13 | | | 0.93 | | | 1.43 | | | 0.88 | |
| 10 | | North Carolina | | | | 93 | | | 0.96 | | | | 0.91 | | | 1.01 | | | 0.26 | | | 0.12 | | | 0.40 | | | 0.68 | | | 53 | | | 28 | | | 10 | | | 2 | | 0.11 | | | 0.99 | | | 1.31 | | | 0.17 | |
| 11 | | Oregon | | | | 93 | | | 0.89 | | | | 0.81 | | | 0.97 | | | 0.32 | | | 0.17 | | | 0.46 | | | 0.66 | | | 49 | | | 26 | | | 12 | | | 6 | | 0.10 | | | 0.97 | | | 1.30 | | | 0.36 | |
| 12 | | PHI | | | | 93 | | | 0.80 | | | | 0.69 | | | 0.91 | | | 0.66 | | | 0.51 | | | 0.81 | | | 0.74 | | | 44 | | | 13 | | | 25 | | | 11 | | 0.16 | | | 0.97 | | | 2.30 | | | 0.31 | |
| 13 | | RTST | | | | 93 | | | 0.47 | | | | 0.34 | | | 0.60 | | | 0.87 | | | 0.76 | | | 0.98 | | | 0.63 | | | 26 | | | 5 | | | 33 | | | 29 | | 0.41 | | | 0.95 | | | 3.36 | | | 0.61 | |
| 14 | | SWAS | | | | 93 | | | 0.38 | | | | 0.25 | | | 0.51 | | | 0.89 | | | 0.80 | | | 0.99 | | | 0.59 | | | 21 | | | 4 | | | 34 | | | 34 | | 0.31 | | | 0.95 | | | 3.33 | | | 0.70 | |
| 15 | | Trauma Score | | | | 93 | | | 0.16 | | | | 0.07 | | | 0.26 | | | 1.00 | | | 1.00 | | | 1.00 | | | 0.51 | | | 9 | | | 0 | | | 38 | | | 46 | | 1.00 | | | 0.93 | | | 13.23 | | | 0.84 | |
| 16 | | Trauma Scorecard | | | | 93 | | | 0.89 | | | | 0.81 | | | 0.97 | | | 0.42 | | | 0.26 | | | 0.58 | | | 0.70 | | | 49 | | | 22 | | | 16 | | | 6 | | 0.11 | | | 0.98 | | | 1.53 | | | 0.27 | |
| 17 | | TTR | | | | 93 | | | 0.87 | | | | 0.78 | | | 0.96 | | | 0.42 | | | 0.26 | | | 0.58 | | | 0.69 | | | 48 | | | 22 | | | 16 | | | 7 | | 0.11 | | | 0.97 | | | 1.50 | | | 0.32 | |
| 18 | | US Field Triage | | | | 93 | | | 0.93 | | | | 0.86 | | | 1.00 | | | 0.26 | | | 0.12 | | | 0.40 | | | 0.66 | | | 51 | | | 28 | | | 10 | | | 4 | | 0.10 | | | 0.98 | | | 1.26 | | | 0.30 | |
| 19 | | Victoria | | | | 93 | | | 0.93 | | | | 0.86 | | | 1.00 | | | 0.03 | | | -0.02 | | | 0.08 | | | 0.56 | | | 51 | | | 37 | | | 1 | | | 4 | | 0.08 | | | 0.90 | | | 0.96 | | | 2.09 | |
| 20 | | Vittel | | | | 93 | | | 0.93 | | | | 0.86 | | | 1.00 | | | 0.13 | | | 0.02 | | | 0.24 | | | 0.60 | | | 51 | | | 33 | | | 5 | | | 4 | | 0.09 | | | 0.96 | | | 1.07 | | | 0.57 | |
| 21 | | WMAS | | | | 93 | | | 0.93 | | | | 0.86 | | | 1.00 | | | 0.34 | | | 0.19 | | | 0.49 | | | 0.69 | | | 51 | | | 25 | | | 13 | | | 4 | | 0.12 | | | 0.98 | | | 1.41 | | | 0.23 | |
| 22 | | YAS | | | | 93 | | | 0.89 | | | | 0.81 | | | 0.97 | | | 0.50 | | | 0.34 | | | 0.66 | | | 0.73 | | | 49 | | | 19 | | | 19 | | | 6 | | 0.13 | | | 0.98 | | | 1.77 | | | 0.23 | |
| **Higher energy trauma** | | | | | | | | | | | | | | | | | | | | | | | | | | | | | | | | | | | | | | | | | | | | | | | | | | | | |
| **Tool Number** | | **Tool Name** | | | | **n** | | | **Sensitivity** | | | | **LCL** | | | **UCL** | | | **Specificity** | | | **LCL** | | | **UCL** | | | **Accuracy** | | | **TP** | | | **FP** | | | **TN** | | | **FN** | | **PPV** | | | **NPV** | | | **Positive LR** | | | **Negative LR** | |
| 1 | | CRAMS | | | | 906 | | | 0.41 | | | | 0.37 | | | 0.45 | | | 0.90 | | | 0.87 | | | 0.93 | | | 0.59 | | | 232 | | | 33 | | | 307 | | | 334 | | 0.26 | | | 0.95 | | | 4.22 | | | 0.65 | |
| 2 | | Dutch | | | | 906 | | | 0.34 | | | | 0.30 | | | 0.38 | | | 0.85 | | | 0.81 | | | 0.89 | | | 0.53 | | | 193 | | | 51 | | | 289 | | | 373 | | 0.16 | | | 0.94 | | | 2.27 | | | 0.78 | |
| 3 | | Florida | | | | 906 | | | 0.49 | | | | 0.45 | | | 0.54 | | | 0.77 | | | 0.73 | | | 0.82 | | | 0.60 | | | 280 | | | 77 | | | 263 | | | 286 | | 0.16 | | | 0.95 | | | 2.18 | | | 0.65 | |
| 4 | | LAS (current) | | | | 906 | | | 0.52 | | | | 0.48 | | | 0.56 | | | 0.80 | | | 0.76 | | | 0.85 | | | 0.63 | | | 296 | | | 67 | | | 273 | | | 270 | | 0.19 | | | 0.96 | | | 2.65 | | | 0.59 | |
| 5 | | LAS (old) | | | | 906 | | | 0.66 | | | | 0.62 | | | 0.70 | | | 0.74 | | | 0.69 | | | 0.79 | | | 0.69 | | | 374 | | | 88 | | | 252 | | | 192 | | 0.17 | | | 0.96 | | | 2.55 | | | 0.46 | |
| 6 | | MATTS balanced | | | | 906 | | | 0.66 | | | | 0.62 | | | 0.70 | | | 0.76 | | | 0.71 | | | 0.80 | | | 0.70 | | | 375 | | | 83 | | | 257 | | | 191 | | 0.18 | | | 0.97 | | | 2.71 | | | 0.45 | |
| 7 | | MATTS sensitive | | | | 906 | | | 0.82 | | | | 0.79 | | | 0.85 | | | 0.61 | | | 0.56 | | | 0.66 | | | 0.74 | | | 464 | | | 132 | | | 208 | | | 102 | | 0.14 | | | 0.98 | | | 2.11 | | | 0.30 | |
| 8 | | MATTS specific | | | | 906 | | | 0.46 | | | | 0.42 | | | 0.50 | | | 0.89 | | | 0.85 | | | 0.92 | | | 0.62 | | | 260 | | | 38 | | | 302 | | | 306 | | 0.26 | | | 0.95 | | | 4.11 | | | 0.61 | |
| 9 | | MGAP | | | | 906 | | | 0.31 | | | | 0.27 | | | 0.35 | | | 0.93 | | | 0.90 | | | 0.95 | | | 0.54 | | | 176 | | | 25 | | | 315 | | | 390 | | 0.25 | | | 0.94 | | | 4.23 | | | 0.74 | |
| 10 | | North Carolina | | | | 906 | | | 0.52 | | | | 0.48 | | | 0.56 | | | 0.78 | | | 0.74 | | | 0.82 | | | 0.62 | | | 295 | | | 75 | | | 265 | | | 271 | | 0.17 | | | 0.95 | | | 2.36 | | | 0.61 | |
| 11 | | Oregon | | | | 906 | | | 0.46 | | | | 0.42 | | | 0.51 | | | 0.83 | | | 0.79 | | | 0.87 | | | 0.60 | | | 263 | | | 57 | | | 283 | | | 303 | | 0.20 | | | 0.95 | | | 2.77 | | | 0.64 | |
| 12 | | PHI | | | | 906 | | | 0.24 | | | | 0.21 | | | 0.28 | | | 0.91 | | | 0.89 | | | 0.94 | | | 0.49 | | | 137 | | | 29 | | | 311 | | | 429 | | 0.19 | | | 0.94 | | | 2.84 | | | 0.83 | |
| 13 | | RTST | | | | 906 | | | 0.37 | | | | 0.33 | | | 0.41 | | | 0.90 | | | 0.87 | | | 0.93 | | | 0.57 | | | 209 | | | 33 | | | 307 | | | 357 | | 0.25 | | | 0.95 | | | 3.80 | | | 0.70 | |
| 14 | | SWAS | | | | 906 | | | 0.40 | | | | 0.36 | | | 0.44 | | | 0.92 | | | 0.90 | | | 0.95 | | | 0.59 | | | 224 | | | 26 | | | 314 | | | 342 | | 0.30 | | | 0.95 | | | 5.18 | | | 0.65 | |
| 15 | | Trauma Score | | | | 906 | | | 0.12 | | | | 0.10 | | | 0.15 | | | 0.99 | | | 0.99 | | | 1.00 | | | 0.45 | | | 70 | | | 2 | | | 338 | | | 496 | | 0.69 | | | 0.93 | | | 21.03 | | | 0.88 | |
| 16 | | Trauma Scorecard | | | | 906 | | | 0.43 | | | | 0.39 | | | 0.47 | | | 0.84 | | | 0.80 | | | 0.87 | | | 0.58 | | | 244 | | | 56 | | | 284 | | | 322 | | 0.18 | | | 0.95 | | | 2.62 | | | 0.68 | |
| 17 | | TTR | | | | 906 | | | 0.27 | | | | 0.23 | | | 0.30 | | | 0.91 | | | 0.87 | | | 0.94 | | | 0.51 | | | 151 | | | 32 | | | 308 | | | 415 | | 0.20 | | | 0.94 | | | 2.84 | | | 0.81 | |
| 18 | | US Field Triage | | | | 906 | | | 0.52 | | | | 0.48 | | | 0.56 | | | 0.78 | | | 0.73 | | | 0.82 | | | 0.62 | | | 296 | | | 76 | | | 264 | | | 270 | | 0.17 | | | 0.95 | | | 2.34 | | | 0.61 | |
| 19 | | Victoria | | | | 906 | | | 0.73 | | | | 0.69 | | | 0.77 | | | 0.57 | | | 0.52 | | | 0.62 | | | 0.67 | | | 413 | | | 146 | | | 194 | | | 153 | | 0.12 | | | 0.96 | | | 1.70 | | | 0.47 | |
| 20 | | Vittel | | | | 906 | | | 0.56 | | | | 0.52 | | | 0.60 | | | 0.77 | | | 0.73 | | | 0.82 | | | 0.64 | | | 319 | | | 78 | | | 262 | | | 247 | | 0.17 | | | 0.96 | | | 2.46 | | | 0.57 | |
| 21 | | WMAS | | | | 906 | | | 0.55 | | | | 0.50 | | | 0.59 | | | 0.78 | | | 0.74 | | | 0.83 | | | 0.63 | | | 309 | | | 74 | | | 266 | | | 257 | | 0.17 | | | 0.96 | | | 2.51 | | | 0.58 | |
| 22 | | YAS | | | | 906 | | | 0.61 | | | | 0.57 | | | 0.65 | | | 0.74 | | | 0.69 | | | 0.79 | | | 0.66 | | | 343 | | | 88 | | | 252 | | | 223 | | 0.16 | | | 0.96 | | | 2.34 | | | 0.53 | |
| **Ground level falls** | | | | | | | | | | | | | | | | | | | | | | | | | | | | | | | | | | | | | | | | | | | | | | | | | | | | |
| Tool number | Tool name | | | n | TP | | FP | | | TN | | FN | | Sensitivity | | | | Sens LCL | | | Sens UCL | | Specificity | | | | Spec LCL | | | Spec UCL | PPV | | NPV | | | Positive LR | | | Positive LR LCL | | | | Positive LR UCL | | | Negative LR | | | | Negative LR LCL | | Negative LR UCL |
| 1 | CRAMS | | | 1,701 | 78 | | 74 | | | 1,265 | | 284 | | 0.22 | | | | 0.17 | | | 0.26 | | 0.94 | | | | 0.93 | | | 0.96 | 0.05 | | 0.99 | | | 3.90 | | | 2.90 | | | | 5.24 | | | 0.83 | | | | 0.66 | | 1.04 |
| 2 | Dutch | | | 1,701 | 58 | | 62 | | | 1,277 | | 304 | | 0.16 | | | | 0.12 | | | 0.20 | | 0.95 | | | | 0.94 | | | 0.96 | 0.04 | | 0.99 | | | 3.46 | | | 2.47 | | | | 4.86 | | | 0.88 | | | | 0.69 | | 1.13 |
| 3 | Florida | | | 1,701 | 99 | | 162 | | | 1,177 | | 263 | | 0.27 | | | | 0.23 | | | 0.32 | | 0.88 | | | | 0.86 | | | 0.90 | 0.03 | | 0.99 | | | 2.26 | | | 1.81 | | | | 2.82 | | | 0.83 | | | | 0.71 | | 0.97 |
| 4 | LAS (current) | | | 1,701 | 118 | | 109 | | | 1,230 | | 244 | | 0.33 | | | | 0.28 | | | 0.37 | | 0.92 | | | | 0.90 | | | 0.93 | 0.05 | | 0.99 | | | 4.00 | | | 3.17 | | | | 5.06 | | | 0.73 | | | | 0.60 | | 0.89 |
| 5 | LAS (old) | | | 1,701 | 144 | | 150 | | | 1,189 | | 218 | | 0.40 | | | | 0.35 | | | 0.45 | | 0.89 | | | | 0.87 | | | 0.90 | 0.04 | | 0.99 | | | 3.55 | | | 2.92 | | | | 4.32 | | | 0.68 | | | | 0.57 | | 0.81 |
| 6 | MATTS balanced | | | 1,701 | 163 | | 136 | | | 1,203 | | 199 | | 0.45 | | | | 0.40 | | | 0.50 | | 0.90 | | | | 0.88 | | | 0.91 | 0.05 | | 0.99 | | | 4.43 | | | 3.64 | | | | 5.39 | | | 0.61 | | | | 0.51 | | 0.74 |
| 7 | MATTS sensitive | | | 1,701 | 208 | | 275 | | | 1,064 | | 154 | | 0.57 | | | | 0.52 | | | 0.63 | | 0.79 | | | | 0.77 | | | 0.82 | 0.03 | | 0.99 | | | 2.80 | | | 2.44 | | | | 3.21 | | | 0.54 | | | | 0.46 | | 0.63 |
| 8 | MATTS specific | | | 1,701 | 86 | | 43 | | | 1,296 | | 276 | | 0.24 | | | | 0.19 | | | 0.28 | | 0.97 | | | | 0.96 | | | 0.98 | 0.08 | | 0.99 | | | 7.40 | | | 5.23 | | | | 10.47 | | | 0.79 | | | | 0.58 | | 1.06 |
| 9 | MGAP | | | 1,701 | 113 | | 228 | | | 1,111 | | 249 | | 0.31 | | | | 0.26 | | | 0.36 | | 0.83 | | | | 0.81 | | | 0.85 | 0.02 | | 0.99 | | | 1.83 | | | 1.51 | | | | 2.22 | | | 0.83 | | | | 0.72 | | 0.95 |
| 10 | North Carolina | | | 1,701 | 90 | | 106 | | | 1,233 | | 272 | | 0.25 | | | | 0.20 | | | 0.29 | | 0.92 | | | | 0.91 | | | 0.94 | 0.04 | | 0.99 | | | 3.14 | | | 2.43 | | | | 4.06 | | | 0.82 | | | | 0.67 | | 0.99 |
| 11 | Oregon | | | 1,701 | 87 | | 89 | | | 1,250 | | 275 | | 0.24 | | | | 0.20 | | | 0.28 | | 0.93 | | | | 0.92 | | | 0.95 | 0.04 | | 0.99 | | | 3.62 | | | 2.76 | | | | 4.74 | | | 0.81 | | | | 0.66 | | 1.00 |
| 12 | PHI | | | 1,701 | 31 | | 37 | | | 1,302 | | 331 | | 0.09 | | | | 0.06 | | | 0.11 | | 0.97 | | | | 0.96 | | | 0.98 | 0.04 | | 0.99 | | | 3.10 | | | 1.95 | | | | 4.92 | | | 0.94 | | | | 0.68 | | 1.29 |
| 13 | RTST | | | 1,701 | 80 | | 88 | | | 1,251 | | 282 | | 0.22 | | | | 0.18 | | | 0.26 | | 0.93 | | | | 0.92 | | | 0.95 | 0.04 | | 0.99 | | | 3.36 | | | 2.54 | | | | 4.45 | | | 0.83 | | | | 0.68 | | 1.03 |
| 14 | SWAS | | | 1,701 | 76 | | 54 | | | 1,285 | | 286 | | 0.21 | | | | 0.17 | | | 0.25 | | 0.96 | | | | 0.95 | | | 0.97 | 0.06 | | 0.99 | | | 5.21 | | | 3.75 | | | | 7.23 | | | 0.82 | | | | 0.63 | | 1.07 |
| 15 | Trauma Score | | | 1,701 | 22 | | 12 | | | 1,327 | | 340 | | 0.06 | | | | 0.04 | | | 0.09 | | 0.99 | | | | 0.99 | | | 1.00 | 0.09 | | 0.99 | | | 6.78 | | | 3.39 | | | | 13.57 | | | 0.95 | | | | 0.54 | | 1.67 |
| 16 | Trauma Scorecard | | | 1,701 | 74 | | 75 | | | 1,264 | | 288 | | 0.20 | | | | 0.16 | | | 0.25 | | 0.94 | | | | 0.93 | | | 0.96 | 0.04 | | 0.99 | | | 3.65 | | | 2.71 | | | | 4.92 | | | 0.84 | | | | 0.67 | | 1.06 |
| 17 | TTR | | | 1,701 | 42 | | 34 | | | 1,305 | | 320 | | 0.12 | | | | 0.08 | | | 0.15 | | 0.97 | | | | 0.97 | | | 0.98 | 0.05 | | 0.99 | | | 4.57 | | | 2.95 | | | | 7.07 | | | 0.91 | | | | 0.65 | | 1.27 |
| 18 | US Field Triage | | | 1,701 | 105 | | 130 | | | 1,209 | | 257 | | 0.29 | | | | 0.24 | | | 0.34 | | 0.90 | | | | 0.89 | | | 0.92 | 0.04 | | 0.99 | | | 2.99 | | | 2.37 | | | | 3.76 | | | 0.79 | | | | 0.66 | | 0.94 |
| 19 | Victoria | | | 1,701 | 146 | | 276 | | | 1,063 | | 216 | | 0.40 | | | | 0.35 | | | 0.45 | | 0.79 | | | | 0.77 | | | 0.82 | 0.02 | | 0.99 | | | 1.96 | | | 1.66 | | | | 2.30 | | | 0.75 | | | | 0.66 | | 0.86 |
| 20 | Vittel | | | 1,701 | 98 | | 133 | | | 1,206 | | 264 | | 0.27 | | | | 0.22 | | | 0.32 | | 0.90 | | | | 0.88 | | | 0.92 | 0.03 | | 0.99 | | | 2.73 | | | 2.16 | | | | 3.44 | | | 0.81 | | | | 0.68 | | 0.96 |
| 21 | WMAS | | | 1,701 | 116 | | 131 | | | 1,208 | | 246 | | 0.32 | | | | 0.27 | | | 0.37 | | 0.90 | | | | 0.89 | | | 0.92 | 0.04 | | 0.99 | | | 3.28 | | | 2.63 | | | | 4.09 | | | 0.75 | | | | 0.63 | | 0.90 |
| 22 | YAS | | | 1,701 | 133 | | 150 | | | 1,189 | | 229 | | 0.37 | | | | 0.32 | | | 0.42 | | 0.89 | | | | 0.87 | | | 0.90 | 0.04 | | 0.99 | | | 3.28 | | | 2.68 | | | | 4.02 | | | 0.71 | | | | 0.60 | | 0.84 |

CRAMS: Circulation, Respiration, Abdomen, Motor, and Speech Scale; Dutch: Dutch Field Triage Protocol; Florida: State of Florida Trauma Criteria; LAS (current): London Ambulance Service (current) Major Trauma Triage Tool; LAS (old): London Ambulance Service (old) Major Trauma Triage Tool; MATTS balanced: Newly developed MATTS triage tool – balancing sensitivity/specificity; MATTS sensitive: Newly developed MATTS triage tool – prioritising sensitivity; MATTS specific: Newly developed MATTS triage tool – prioritising specificity; MGAP: Mechanism, Glasgow Coma Scale, Age, and Arterial Pressure Score; North Carolina: North Carolina Trauma and Burn EMS Triage and Destination Plan; Oregon: Oregon Guidelines for Field Triage of Injured Patients; PHI: The Prehospital Index; RTST: Triage Revised Trauma Score; SWAS: South West Ambulance Service Major Trauma Triage Tool; Trauma Score: Trauma Score; Trauma Scorecard: Trauma Scorecard; TTR: Trauma Triage Rule; US Field Triage: National Guidelines for the Field Triage of Injured Patients (2011); Victoria: Pre-hospital Major Trauma Triage - Trauma Victoria; Vittel: Vittel criteria for severe trauma triage; WMAS: West Midlands Ambulance Service Major Trauma Triage Tool: YAS Yorkshire Ambulance Service Major Trauma Triage Tool
