# Supplementary material for: Major Trauma Triage Study (MATTS): Diagnostic accuracy of major trauma triage tools in English regional trauma networks – A case-cohort study
Source: PLoS One. 2026 Mar 27;21(3):e0344996. doi: 10.1371/journal.pone.0344996 (PMC13029787; doi:10.1371/journal.pone.0344996)
Supplement: S6 Table — (DOCX) [file pone.0344996.s006.docx]

**SUPPLEMENTARY MATERIALS S6**

**QUADAS-2 Internal and external validity assessment**

| **CRITICAL APPRAISAL / RISK OF BIAS ACCORDING TO QUADAS-2 CRITERIA** | | | |
| --- | --- | --- | --- |
| **QUADAS 2 Domain** | **Potential source of bias** | **Risk of bias rating** | **Comments/mitigation** |
| **Patient selection** | Selection bias arising from non-consecutive or non-random sampling | Low | ▪Inclusion of consecutive eligible ambulance service patients |
|  | Selection bias arising from case-control design | Low | ▪Single gate case-cohort design |
|  | Selection bias from inappropriate exclusions | Low | ▪Inclusion criteria targeting patients where triage tool would plausibly be used  ▪Exploration of spectrum effects in subgroups with higher energy injury mechanisms. |
|  | Selection bias from non-paired comparative accuracy design | Low | ▪All triage tools evaluated in same patient sample |
|  |  |  |  |
| **Index test** | Test review bias | Low | ▪Data coders blinded to reference standard status  ▪Statistical coding of triage tools performed blinded to reference standard status, with quality control by independent statistician. |
|  | Bias from selecting test threshold to optimise sensitivity/specificity | Low | ▪Prediction models assessed at pre-published recommended cut-points |
|  | Information bias from conduct or interpretation of the index test | Moderate | ▪Triage tools evaluated using routinely collected ambulance service data, with possibility of erroneous, missing, or inaccurate data leading to index test misclassification. |
|  | Undergoing one index test likely to affect the performance of the other index test(s) | Low | ▪Coding of individual triage tools independently performed |
|  |  |  |  |
| **Reference standard** | Reference standard misclassification | Unclear | ▪Reference standard coded for TARN cases using routinely collected hospital data, with possibility of erroneous, missing, or inaccurate data.  ▪Possibility of non-identification of reference standard positive cases due to TARN inclusion criteria e.g. penetrating trauma patient discharge <72 hours.  ▪Possibility of non-enrolment/submission of reference standard positive cases to TARN, especially in trauma units not paid for case submissions (mitigated by selection of high performing trauma networks)  ▪Possibility for reference standards including interventions (e.g., interventional radiology) that treatment was not received if patient was transported to a trauma unit, rather than a MTC, due to lack of availability, rather than non-requirement. Partially mitigated by inclusion of 'hot' transfer to MTC within 6 hours of presentation as positive criterion in reference standard.  ▪Possibility of delayed enrolment/submission of reference standard positive cases to TARN due post-mortems (mitigated by final TARN data transfer 9 months after study inclusion dates)  ▪ Inaccurate matching of TARN and ambulances service records. Very small proportion of reference standard positive cases not matched to a corresponding ambulance service record (<5%). However, it is likely that these represent non-participating ambulance service cases. Also, there is a low probability that a genuinely unmatched ambulance service record would be randomly sampled as a 'non-case' (<5%). |
|  | Diagnostic review bias | Low | ▪Reference standard coded independently by TARN without knowledge of index test status. |
|  | Incorporation bias | Low | ▪Reference standard classification independent of index test information |
|  |  |  |  |
| **Patient flow** | Partial verification bias | Low | ▪All patients had potential for TARN submission and reference standard coding, regardless of triage status. |
|  | Differential verification bias | Low | ▪Triage tool positive and negative patients received the same reference standard classification procedures |
|  | Selection bias from loss to follow up or missing data | Low | ▪Complete case main analysis performed with potential for bias if missing data not missing completely at random  ▪However, sensitivity analyses demonstrate that results are robust to missing at random, or extreme missing not at random, assumptions. |
|  | Different reference standards used for different tests | Low | ▪All triage tools assessed with same primary and secondary reference standards |
|  | Selection bias from differential loss to follow up or missing data across different triage tools | Low | ▪Missing data levels similar across triage tools (as most triage tools include similar physiology)  ▪Sensitivity analyses demonstrate that results are robust to missing at random, or extreme missing not at random, assumptions. |
|  | Disease progression bias | Low | ▪Reference standard classification performed from time of injury to defined time points |
| **GENERALISABILITY OF FINDINGS ACCORDING TO QUADAS-2 CRITERIA** | | | |
| **QUADAS 2 Domain** | **Source of applicability concerns** | **Applicability concern rating** | **Comments** |
| **Patient selection** | Any concerns that included patients do not correspond to the research question | Low | ▪Assumption that triage tool should be applied to all patients presenting with acute non-trivial injuries.  ▪Exploration of spectrum effects in subgroups with higher energy injury mechanisms.  ▪Selected working impression codes may not fully represent acute non-trivial injury patients where a triage tool would be used. However, sensitivity analysis including reference standard positive cases with non-selected working impression codes had negligible effect on sensitivity results.  ▪Only patients from predominant ambulance service serving each trauma network included. Patients from neighbouring ambulances services, transported to included trauma network hospitals excluded. |
| **Index test** | Concerns that the index test, its conduct or its interpretation differ from the review question | Moderate | ▪Theoretical performance of triage tools investigated with index tests coded according to recorded prehospital data.  ▪Real life performance of triage tools in participating ambulance services evaluated in separate study. |
| **Reference standard** | Concerns that the target condition as defined by the reference standard does not match the review question. | Low | ▪Reference standard developed by consensus to reflect major trauma benefiting from direct MTC care in the NHS  ▪Alternative secondary reference standards also evaluated |
